# Fine Mapping and Evolution of the Major Sex Determining Region in Turbot (*Scophthalmus maximus*)

**DOI:** 10.1534/g3.114.012328

**Published:** 2014-08-07

**Authors:** Xoana Taboada, Miguel Hermida, Belén G. Pardo, Manuel Vera, Francesc Piferrer, Ana Viñas, Carmen Bouza, Paulino Martínez

**Affiliations:** * Departamento de Genética, Facultad de Biología (CIBUS), Universidad de Santiago de Compostela, Avda. Lope Gómez de Marzoa, 15782 Santiago de Compostela, Spain; † Departamento de Genética, Facultad de Veterinaria, Universidad de Santiago de Compostela, Campus de Lugo, 27002 Lugo, Spain; ‡ Departament of Biology, Faculty of Sciences, University of Girona, Campus de Montilivi s/n, 17071 Girona, Spain; § Institut de Ciènces del Mar, Consejo Superior de Investigaciones Científicas (CSIC), Barcelona, Spain

**Keywords:** turbot, sex determining master gene, comparative mapping, sex genetic differentiation, evolution of sex determination, genetics of sex

## Abstract

Fish sex determination (SD) systems are varied, suggesting evolutionary changes including either multiple evolution origins of genetic SD from nongenetic systems (such as environmental SD) and/or turnover events replacing one genetic system by another. When genetic SD is found, cytological differentiation between the two members of the sex chromosome pair is often minor or undetectable. The turbot (*Scophthalmus maximus*), a valuable commercial flatfish, has a ZZ/ZW system and a major SD region on linkage group 5 (LG5), but there are also other minor genetic and environmental influences. We here report refined mapping of the turbot SD region, supported by comparative mapping with model fish species, to identify the turbot master SD gene. Six genes were located to the SD region, two of them associated with gonad development (*sox2* and *dnajc19*). All showed a high association with sex within families (*P* = 0), but not at the population level, so they are probably partially sex-linked genes, but not SD gene itself. Analysis of crossovers in LG5 using two families confirmed a ZZ/ZW system in turbot and suggested a revised map position for the master gene. Genetic diversity and differentiation for 25 LG5 genetic markers showed no differences between males and females sampled from a wild population, suggesting a recent origin of the SD region in turbot. We also analyzed associations with markers of the most relevant sex-related linkage groups in brill (*S. rhombus*), a closely related species to turbot; the data suggest that an ancient XX/XY system in brill changed to a ZZ/ZW mechanism in turbot.

Gonad differentiation is an excellent example of developmental plasticity (Siegfried 2010). In vertebrates, two highly differentiated and specialized gonads, testes and ovaries, develop from a single undifferentiated primordium by a well-defined pathway. A binary decision at the beginning of development often is controlled by specific sex-determining genes (SDg). A general consensus existed until recently that the genetic cascade/network underlying this process should be highly conserved and differences would only occur at its top (Marín and Baker 1998; Charlesworth and Mank 2010).

The evolutionary origin of heteromorphic sex chromosome pairs is thought to be due to the evolution of a nonrecombining region that occurs after a sexually antagonistic gene (with one allele favorable in one sex but detrimental in the other, often termed “SA”) establishes a polymorphism in a genome region linked to a new SDg (reviewed by Mank and Avise 2009; Charlesworth and Mank 2010). This situation favors suppressed recombination in the region, leading, over time, to the accumulation of recessive, deleterious mutations and repetitive DNA on the SDg-bearing chromosome, often termed “genetic degeneration” (reviewed by Charlesworth and Charlesworth 2000), and ultimately, via chromosome rearrangements and deletions, to the heteromorphic sex chromosomes characteristic of mammals, birds, or *Drosophila* (Charlesworth *et al.* 2005).

Initial data from fish, however, found a different picture (Devlin and Nagahama 2002; Schartl 2004). Although SA genes are known in fish species, mostly related to color differences associated with courtship (Roberts *et al.* 2009; Tripathi *et al.* 2009; Parnell and Streelman 2013), most fish species do not have heteromorphic sex chromosomes (Penman and Piferrer 2008), and fish genetic SD systems are thought to involve sex chromosomes in an early evolutionary stage (Piferrer *et al.* 2012). This finding suggests a high evolutionary turnover rate of SDg. Furthermore, genetic differences have been observed not only at the top but also downstream of the gonad differentiation cascade in fish (Böhne *et al.* 2013; Herpin *et al.* 2013) and, in addition, more than one sex-related segregating gene and environmental cues have been documented in several species, suggesting that sex determination (SD) in fish can behave as a quantitative trait (Vandeputte *et al.* 2007; Otake *et al.* 2008; Ser *et al.* 2010; Shinomiya *et al.* 2010; Liew *et al.* 2012; Parnell and Streelman 2013).

The flatfish group underwent a rapid evolutionary radiation around 35 million years ago, making relationships among flatfish families controversial (Pardo *et al.* 2006). The monophyletic origin of the group also has been questioned (Campbell *et al.* 2013). Flatfish include species highly valuable for fisheries and aquaculture (Cerdá and Manchado 2013). The SD system has so far been studied in 14 flatfish species, and ZZ/ZW and XX/XY systems both exist in similar proportions (Viñas *et al.* 2013). Although environmental cues have been reported in some species (Yamaguchi *et al.* 2010; Mankiewicz *et al.* 2013), genetic factors are likely the main factors underlying SD in this group (Viñas *et al.* 2013).

Turbot (*Scophthalmus maximus*) is a flatfish mostly cultured in Europe and PR China (Fao 2013). Sexual dimorphism in growth is among the greatest within fish, making it important to understand the SD mechanism, as this might allow development of hormone-free methods to produce monosex populations for turbot culture (Imsland *et al.* 1997; Piferrer *et al.* 2004). A genomic region on linkage group 5 (LG5) segregates as a major ZZ/ZW system, and minor genetic factors have been detected by quantitative trait loci (QTL) screens on LG6, LG8, and LG21 (Martínez *et al.* 2009; Hermida *et al.* 2013). Minor environmental effects also have been reported (Haffray *et al.* 2009). Martínez *et al.* (2009) located the turbot master SD gene 2.6 cM from the LG5 marker with the greatest sex-association (SmaUSC-E30). This sequence was originally identified from a nonannotated, 389-bp turbot expressed sequence tag (EST; FE946656) from an enriched immune-related EST database (Pardo *et al.* 2008) and proved to be part of the 3′ untranslated region of the 1075-bp fragile X mental retardation, autosomal homolog 1 (*fxr1*) gene (KJ434937, BLASTn E-value 3^-61^) in the updated turbot EST database enriched for the gonad-brain axis transcriptome (Ribas *et al.* 2013). Mapping of candidate genes and mining through comparative genomics yielded sex-related genes linked to the reported QTL, giving additional support to the genomic regions in the other LGs (Viñas *et al.* 2012; Hermida *et al.* 2013). Comparative mapping with the brill, a closely related species (*S*. *rhombus*), contributed to understanding of the genetic architecture of growth-related traits (Hermida *et al.* 2014) but was not extended to studying SD. Finally, functional genomics studies have identified genes differentially expressed in male and female turbot (Taboada *et al.* 2012) and revealed the gonad-brain axis genes most relevant for gonad differentiation (Ribas *et al.* 2013). The identity of the SD master gene, and how it interacts with the minor genetic and environmental factors, however, remains elusive.

In this study, we applied the updated version of the turbot map (Hermida *et al.* 2013) to refine gene mapping at the main SD region (Martínez *et al.* 2009) to identify the SDg and to perform population genetic analyses to validate the SD region in this species. Our work included: (i) classical and physical mapping of genes (identified through comparative genomics with model fish) at the main SD region; (ii) analysis of recombination frequencies (RFs) in LG5 (the main SD LG) in males and females; and (iii) study of LG5 markers in a sample from a natural population to identify any chromosome region differentiated between the sexes, and a preliminary association study in brill (*S*. *rhombus*). The results suggest that this region evolved recently.

## Materials and Methods

### Reference families for segregation analysis and mapping

The reference families used for mapping and segregation analysis were those used by Bouza *et al.* (2007, 2012) and Hermida *et al.* (2013). To summarize, HF is a family of haploid gynogenetic embryos obtained at the Instituto Español de Oceanografía (Vigo, Spain) following Piferrer *et al.* (2004). DF and QF are F2 families with known linkage phase obtained from the genetic breeding programs of the Stolt Sea Farm SA and Insuiña SL companies and originating from genetically divergent grandparents sampled from wild populations from the Atlantic area. More details of the haploid (HF) and diploid (DF) families, and the additional seven families used for QTL screening (QF1−QF7), are in Bouza *et al.* (2007) and Sánchez-Molano *et al.* (2011), respectively.

### Natural populations of turbot (*S. maximus*) and brill (*S. rhombus*)

The Stolt Sea Farm SA (SSF) broodstock, representative of a wild Atlantic population of turbot (Martínez *et al.* 2009; Vera *et al.* 2011), was used to test for associations of markers with sex (gender) and to estimate population genetic parameters for genes in the main SD LG. For this, 96 individuals (48 males and 48 females) were used.

Statistical association with sex also was tested in a wild population of brill, a closely related species to turbot (Hermida *et al.* 2014), using the brill broodstock of Agua del Pino experimental aquaculture station (IFAPA), which was founded with wild individuals from Bahía de Cádiz (SW Spain).

### Comparative mapping and gene mining

**Figure 1:**
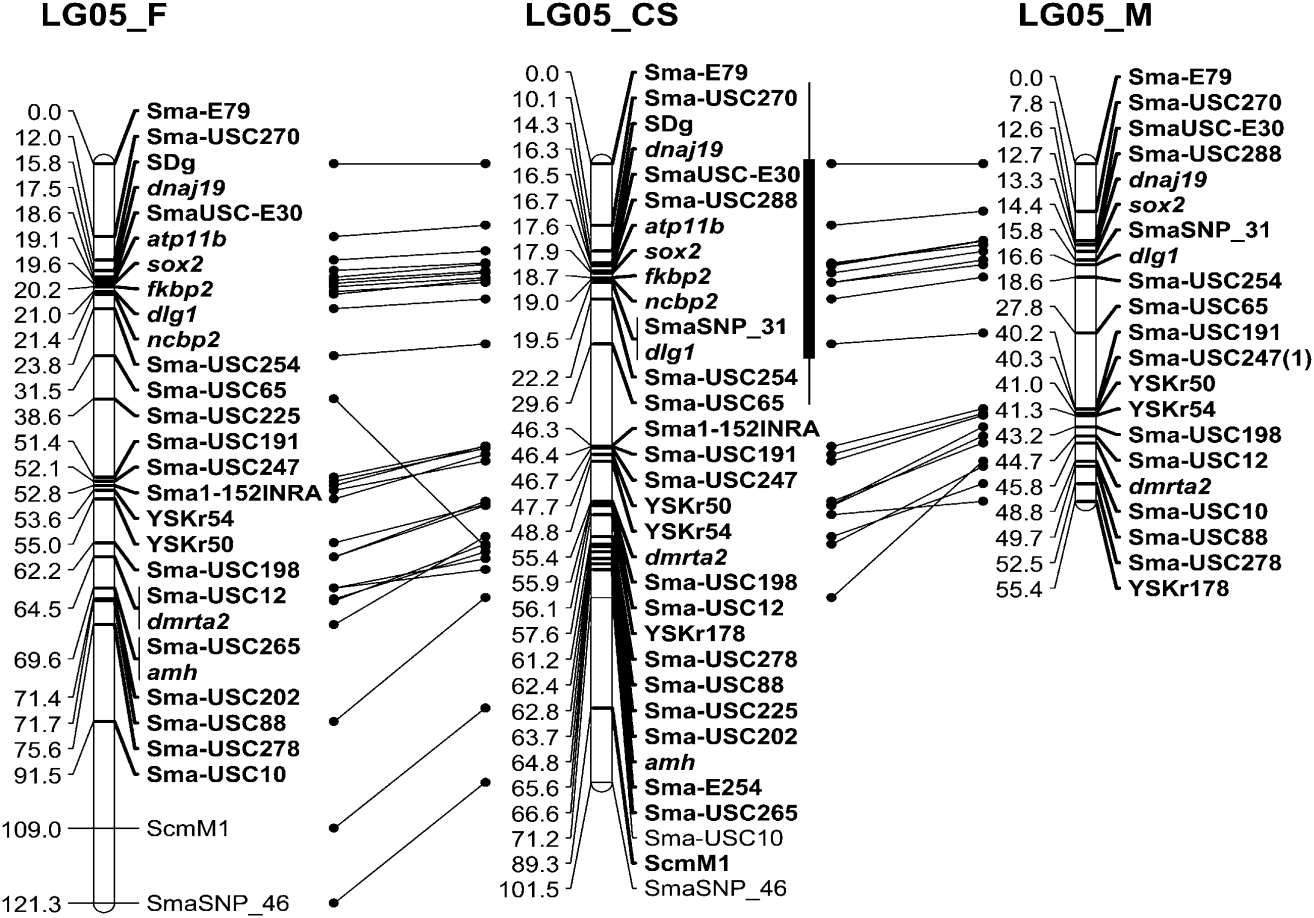
Female (F), male (M), and consensus (SC) turbot (*S. maximus*) LG5 maps. The vertical bar in the consensus map indicates the main sex determination region according to Martínez *et al.* (2009; thin line) and Hermida *et al.* (2013; thick line).

The turbot main SD region was previously localized at the proximal end of LG5, very close to SmaUSC-E30, between Sma-USC270 and Sma-USC65 (Martínez *et al.* 2009) (see Figure 1). New mapping data with additional LG5 markers enabled us to narrow down the SD region to between SmaUSC-E30 and SmaSNP_31 (separated by 5.3 cM; Bouza *et al.* 2012) according to the estimated mapping position of SDg (2.6 cM from SmaUSC-E30; Martínez *et al.* 2009). Previous comparative mapping between turbot and model fish species showed the greatest proportion of homologous sequences and syntenic markers with stickleback (*Gasterosteus aculeatus*; Bouza *et al.* 2012) in accordance with recent phylogenetic data (Zou *et al.* 2012), so its genome was used for gene mining to discover other genes in this region of turbot. Three other Acanthopterygii, medaka (*Oryzias latipes*), fugu (*Fugu rubripes*), and its close relative Tetraodon (*Tetraodon negroviridis*), also were used to study gene order in the turbot SD region. Sequences of LG5 markers were compared against model fish genomes with the use of the National Center for Biotechnology Information Basic Local Alignment Search Tool (*i.e.*, NCBI-BLAST). The list of genes at the region of stickleback LGVIII between homologous sequences of SmaUSC-E30 and SmaSNP_31 was obtained with the BioMart data mining tool (www.ensembl.org). Their transcripts were compared by BLASTn against the turbot transcriptome database (Ribas *et al.* 2013) for further gene marker development and mapping. Those transcripts that exhibited high homology (E-value <10^−50^) were selected for mapping.

### Marker development for mapping

The selected transcripts in the turbot transcriptome database were aligned with the corresponding stickleback sequences (www.ensembl.org) by CLUSTALW (http://www.ebi.ac.uk/Tools/clustalw2/index.html) to estimate the intron positions. Primers to amplify introns were then designed using sequences in the adjacent exons, or in untranslated regions, using Primer3 (http://primer3.ut.ee/). Several amplicons were selected for polymerase chain reaction (PCR) and sequencing in a sample of five males and five females to search for polymorphisms suitable for mapping these genes.

Genomic DNA was extracted from muscle tissue from all individuals to be genotyped, using standard phenol–chloroform procedures (Sambrook *et al.* 1989). PCRs were carried out in a volume of 50 μL, 75 ng of genomic DNA, 20 pmol of each primer, 0.2 mM of each dNTP, 1× PCR buffer, and 2.5 U of GreenTaq DNA polymerase (GenScript). PCR was performed in a MyCycler Thermal cycler (Bio-Rad) following an initial denaturation step at 94° for 3 min; 30 amplification cycles including denaturation at 94° for 30 sec, primer annealing at 60° for 60 sec, and an extension step depending on amplicon size (about 1 kb/min) at 72°; and a final extension step at 72° for 7 min. The PCR products were analyzed on agarose gels stained with SYBR Gold (Invitrogen) and purified with DNA Clean & Concentrator kit (Zymo Research) following the manufacturer’s protocol.

Amplicons were sequenced following the ABI Prism BigDye Terminator v3.1 Cycle Sequencing Kit protocol on an ABI 3730xl Genetic Analyzer (Applied Biosystems). Sequences were aligned and analyzed with SeqScape v2.5 (Applied Biosystems) to identify polymorphisms. Several single-nucleotide polymorphisms (SNPs) were identified in each gene, and variants were selected for mapping based on the allele frequencies and sequencing quality. Primers for SNP genotyping were designed with Primer3. Genotyping reactions were performed in a multiplex PCR following the protocol of Vera *et al.* (2011).

### Mapping

Previous genotyping data from Hermida *et al.* (2013) and the new genotypes obtained here for markers in the main SD region were used for mapping. Informativeness of the new markers was checked by genotyping parents and grandparents of the mapping family panel. Family DF was mapped for many markers (Hermida *et al.* 2013), and thus, it was the main family for our map analyses. QF families were used only when markers were noninformative in DF. We did not genotype the HF family, but we used previous mapping information to obtain the male, female, and consensus maps. In the DF, between 85 and 96 offspring were genotyped for mapping, using an ABI 3730xl DNA sequencer and Genemapper 4.0 (Applied Biosystems). Segregation at each locus was tested for Mendelian proportions using χ^2^ tests, with Bonferroni correction for multiple tests (α = 0.05). Linkage analysis was performed as described by Bouza *et al.* (2012) and Hermida *et al.* (2013) using Joinmap 3.0. Graphics were generated with Mapchart 2.2 (Voorrips 2002).

Physical mapping of candidate genes was performed using fluorescence *in situ* hybridization (FISH) with bacterial artificial chromosome (BAC) probes. The turbot BAC library was generated at the Clemson University Genomics Institute under sponsorship from ReGABA (Galician Net of Biotechnology in Aquaculture; see Taboada *et al.* 2014). To isolate BAC clones carrying putative genes at the main SD region to be used as probes for BAC-FISH, the library was screened with the primers generated for mapping candidate genes via the use of 3-dimensional pooling PCR.

Chromosomal preparations for FISH were obtained from anterior kidney cells following Bouza *et al.* (1994) using turbot fry (90 g) supplied by Cluster de Acuicultura de Galicia (CETGA). The two positive BAC clones (Sma51C11 and Sma58H5) including candidate genes were labeled with digoxigenin-11-dUTP and biotin-16-dUTP (Roche Applied Sciences), respectively, using whole-genome amplification kits, WGA2 and WGA3 (Sigma-Aldrich), according to the supplier’s protocol. FISH was carried out using single and double fluorescence labeling according to Taboada *et al.* (2014). Images were captured with an Olympus BX51 microscope, equipped with an Olympus DP71 color digital camera, and processed with Adobe Photoshop 3.0.

### RF and crossing-over in SD LGs

To increase the precision of RF for LG5, and especially for the main SD region, compared with previous estimates (Bouza *et al.* 2012; Hermida *et al.* 2013), we genotyped all parents of the eight available diploid mapping families (DF, QF1-QF7) for the 12 markers located between Sma-USC270 and Sma-USC65 by Martínez *et al.* (2009), the region that showed the greatest statistical associations with gender within families. Informative markers segregating in the male or female parent in at least one family were selected for further offspring genotyping and RF analysis of marker intervals along LG5 that are common to the different families.

Two mapping families (DF and QF6) with sexed progenies were used for detailed analysis of crossing-over events on LG5, using only framework mapped markers.

We also were interested in evaluating differences in RF between males and females in two candidate LGs related to SD in brill. Because no families were available in this species, we comparatively analyzed linkage disequilibrium in males and females from the IFAPA broodstock using probability tests under the default parameters of GENEPOP 4.2 (http://kimura.univ-montp2.fr/~rousset/Genepop.htm). This approach would permit to detect RF differences between sexes in the putative SD LG if crossing-over were blocked over a chromosome stretch in one of both sexes.

### Population genetics of the major SD-bearing LG (LG5)

Genetic diversity and divergence between males and females was performed with the use of 28 homogeneously distributed genetic markers along LG5 in the SSF turbot broodstock representative of a natural population of Atlantic origin. Genotyping information from Martínez *et al.* (2009; three loci) was combined with data on 25 new LG5 loci genotyped in a sample of 48 males and 48 females of the SSF broodstock. Departures from Hardy–Weinberg genotype proportions were checked by exact tests and the deviations at each locus were quantified by F_IS_ statistics. Genetic differentiation between male and female subsamples was estimated by using F_ST_ and tested using exact probability homogeneity tests.

To estimate genetic diversity, expected heterozygosity (H_e_) and mean number of alleles per locus (A) were computed for all markers and for the microsatellites (most of our markers), because their mutation process differs from that for SNPs. These analyses were implemented using the default options of GENEPOP 4.2 either in the whole sample to compare differences across genomic regions, or in the male and female subsamples to check for allele or genotype frequency differences between sexes.

### Sex association at family and population levels

Contingency χ^2^ tests were conducted to search for genotypic and allelic association between markers and sex within the turbot families, DF and QF6, and in the SSF population sample. Markers from the most suggestive sex-associated LGs of turbot (LG5 and LG21; Hermida *et al.* 2013) also were tested in the brill IFAPA broodstock population, as well as F_ST_ tests for genetic differentiation between males and females. Bonferroni corrections were used for all analyses with multiple tests.

## Results

### Identification and mapping of candidate genes in the major SD region

In the homologous syntenic region of the threespine stickleback LGVIII to the main SD region of turbot (between SmaUSC-E30 and SmaSNP_31; Hermida *et al.* 2013), we found 19 genes, 13 of them functionally annotated (see Supporting Information, Table S1). Thirteen of these genes showed homology (BLASTn E-value < 10^−6^) with sequences in the turbot EST database, including several annotated genes (*dnajc19*, *atp11b*, *fkbp2*, *ncbp2*, *sox2*; Ribas *et al.* 2013) and one anonymous (Ensembl: ENSGACG00000006216) annotated by BLASTn as Disks large homolog 1 (*dlg1*) (E-value < 10^−50^). To map these six genes, PCR primers were designed, and at least one SNP could be consistently genotyped in the amplicons of each gene (see Table 1 and Table S2). These markers were informative in at least one family and were used to construct our new consensus LG5 map (Figure 1). The six genes were located in the proximal region of this map, in a narrow region of 3.2 cM mostly between SmaUSC-E30 and SmaSNP_31, in a similar order to that of the stickleback LGVIII (see Table S1).

**Table 1:** Candidate genes and SNP markers at the major sex determining region of turbot (through comparative mapping against the stickleback LGVIII chromosome

Gene	Stickleback Genome Position, bp	Accession No.	External Primers	Internal Primer	Amplicon Size, pb	Marker Position
*dnaj19*	6068380 - 6070562	KJ434933	F: GCCGTGAAGCAGATGGAG	F: CCACCGGTGATAGTTGTGG	392	Third intron
			R: GGGAAACAATCAATGGATCA			
*sox2*	6157588 - 6158556	KJ434936	F: AGGAAAGTCTCCTGGAAGGAA	R: GTCCCTTTTTCTTTCCAATGTG	662	3′ UTR
			R: CAGATGAAAAGTGGGAGACG			
*atp11b*	6308502 - 6339588	KJ434932	F: AGACTCATTTCTGGACGTGGA	F: GTGGACATGCAGTAGAATAACTGG	370	30th intron
			R: CACCACGTCGGGAAAGAG			
*dlg1*	6549844 - 6552127	KJ434935	F: CAGGAAGAGACTCTGCTCACC	R: TCTTTAAATCCACACTGGGTGATAC	370	Third intron
			R: GAATGGAAGTTTGACGTTGGA			
*fkbp2*	6565695 - 6568855	KJ434934	F: CGAGAAGAGGAAGCTCGTCA	F: TTCCCCAAGTTCTGACTTTGAG	334	Intron
			R: TTGGATGGAGCAAATCTACTGA			
*ncbp2*	6569593 - 6572048	KJ434931	F: GCGTTGATCAGCGACTCCTA	R: CCGTGTTTGCTAACGGCT	352	First intron
			R: GCAATGAGTCCGAACACAAA			

SNP, single-nucleotide polymorphism; LG, linkage group; UTR, untranslated region.

Male and female maps also were constructed using segregation data from the mother and the father of the mapping panel family. Most markers were collinear in all three turbot maps, except for Sma-USC225 (Figure 1). With the added markers, the male map was roughly half the length of the female map, as previously reported (Bouza *et al.* 2012).

**Figure 2:**
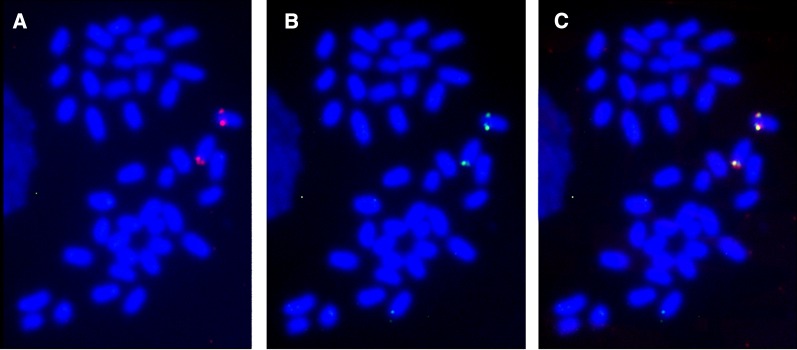
Two-color fluorescence *in situ* hybridization (FISH) using bacterial artificial chromosome (BAC) clones at the main sex determination region of turbot (*S. maximus*). The *fkbp2* and *dlg1*–bearing BAC clone (Sma51C11) and the *ncbp2*–bearing BAC clone (Sma58H5) were labeled with digoxigenin (red) and biotin (green), respectively. (A) Sma51C11; (B) Sma58H5; and (C) double-label BAC-FISH with Sma51C11 and Sma58H5.

The physical position of these genes was studied by BAC-FISH, including double fluorescence labeling. Two BAC clones (Sma51C11 and Sma58H5, around 100 kb each) were identified, one containing the *fkbp2* and *dlg1* genes (Sma51C11), and the other with *ncbp2* (Sma58H5). Both probes cohybridized in the proximal region of a chromosome whose size and morphology correspond to LG5 (Taboada *et al.* 2014), supporting the genetic mapping (Figure 2).

### RFs on LG5

**Figure 3:**
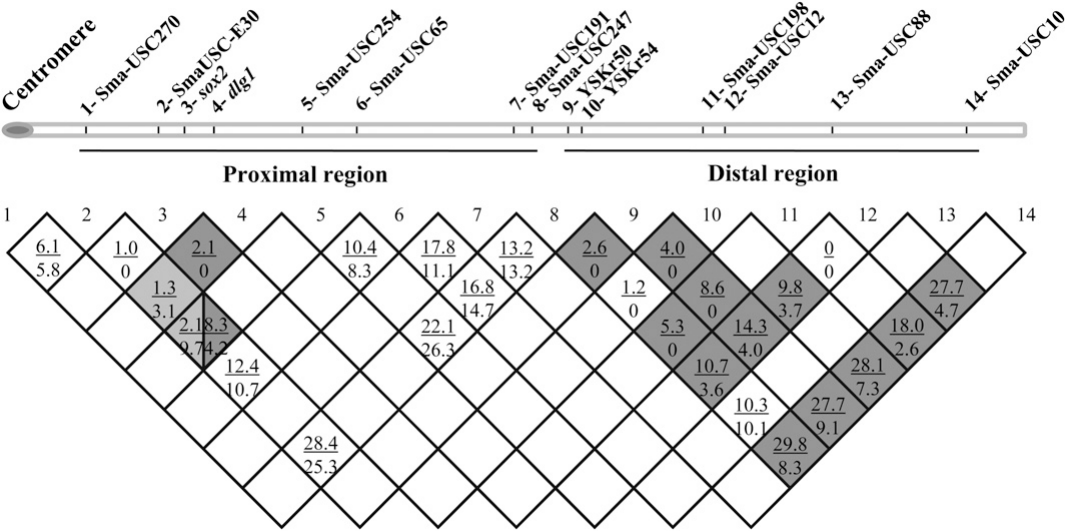
Comparison of recombination frequency (males *vs.* females) along turbot (*S. maximus*) linkage group 5. Above and underlined in each cell recombination frequency in females and below in males.

We combined segregation data for adjacent markers in LG5 from previous reports (Hermida *et al.* 2013) and our new work for the nine mapping families (HF, DF, and QF1−QF7). Information for a given interval generally was available from only a single family, but data for 14 markers identified consistent differences along LG5 (Figure 3). The RF over common intervals in females was greater than in males (f/m ratio 1.8:1, slightly greater than the 1.6:1 observed for the whole turbot map; Bouza *et al.* 2012). A major difference, however, was observed between the proximal (36.2 cM) and distal (23.4 cM) regions (Figure 3). In the proximal region, the average RF for all marker pairs, weighted by the map lengths, was identical in the two sexes, but in the distal region it was nearly four times greater in females (3.6:1). Within the proximal region, differences between males and females, and between different families, were observed within the main SD region (Figure 1 and Figure 3), whereas the remaining intervals recombined at similar rates in both sexes. In the distal region, a first subregion (between YSKr50 and Sma-USC198) showed no recombination in males, whereas in females recombinants were found for all marker pairs, and, in the terminal subregion, the RF was much greater in females than in males. These differences between sexes may be due to chromosome rearrangements as suggested previously (Martínez *et al.* 2009), but the information is not yet sufficient to be conclusive.

### Crossing-over evaluation on the turbot LG5 using sexed families

The fine analysis of crossing-over events in LG5 using framework markers from the sexed DF and QF6 families provided valuable information to narrow the position of the SDg (see Figure S1). Our three generation pedigrees allowed us to infer the genotypes of markers on the Z and W chromosomes in the mothers. The marker order was mostly congruent with the previously mapping information (Figure 1), except for Sma-USC225, whose estimated position required several double recombinants in a small region to explain our observations. After excluding this locus, single and nonrecombinant gametes were found in similar proportions in both families (50% and 46% averaged over families, respectively). Double recombinants were detected only in family DF where many more markers were available (Figure 3). As previously reported (Martínez *et al.* 2009), only those markers segregating in the mother were associated with the sex of progenies, confirming a ZZ/ZW system. Also, the region whose genes have alleles most strongly associated with sex was located at the proximal region of LG5 that includes the SmaUSC-E30 marker (in the *fxr1* gene) but also the closely linked *sox2*, *atp11b*, *fkbp2*, and *dlg1* markers from this study.

The most compatible region between the phenotypic sex and the genetic constitution of the Z (in males) and W (in females) chromosomes coming from the mother was close to SmaUSC-E30 in the selected interval (between SmaUSC-E30 and SmaSNP_31), but it cannot be discarded that it is located between SmaUSC-E30 and SmaUSC-E79. In fact, using all segregation data in DF family and assuming full penetrance and a ZZ/ZW system, the SDg was mapped out of the screened interval at 15.8 and 14.3 cM in the female and the consensus maps, respectively, between SmaUSC-E30 and Sma-E79 (Figure 1). This estimation, however, should be taken with caution because of the assumptions, and, additionally, because there are few genetic markers between SmaUSC-E30 and Sma-E79 that could introduce a bias for mapping.

Several discordances were detected between phenotypic and genetic sex strongly suggesting other genetic or environmental factors involved in SD in turbot. Most discrepancies are related to males with female genotypes including two offspring in DF family (M4 and M30) and in five QF6 family (M14, M24, M25, M31, and M42; Figure S1), which suggests incomplete penetrance of the female genotype.

### Genetic diversity and differentiation along LG5

**Figure 4:**
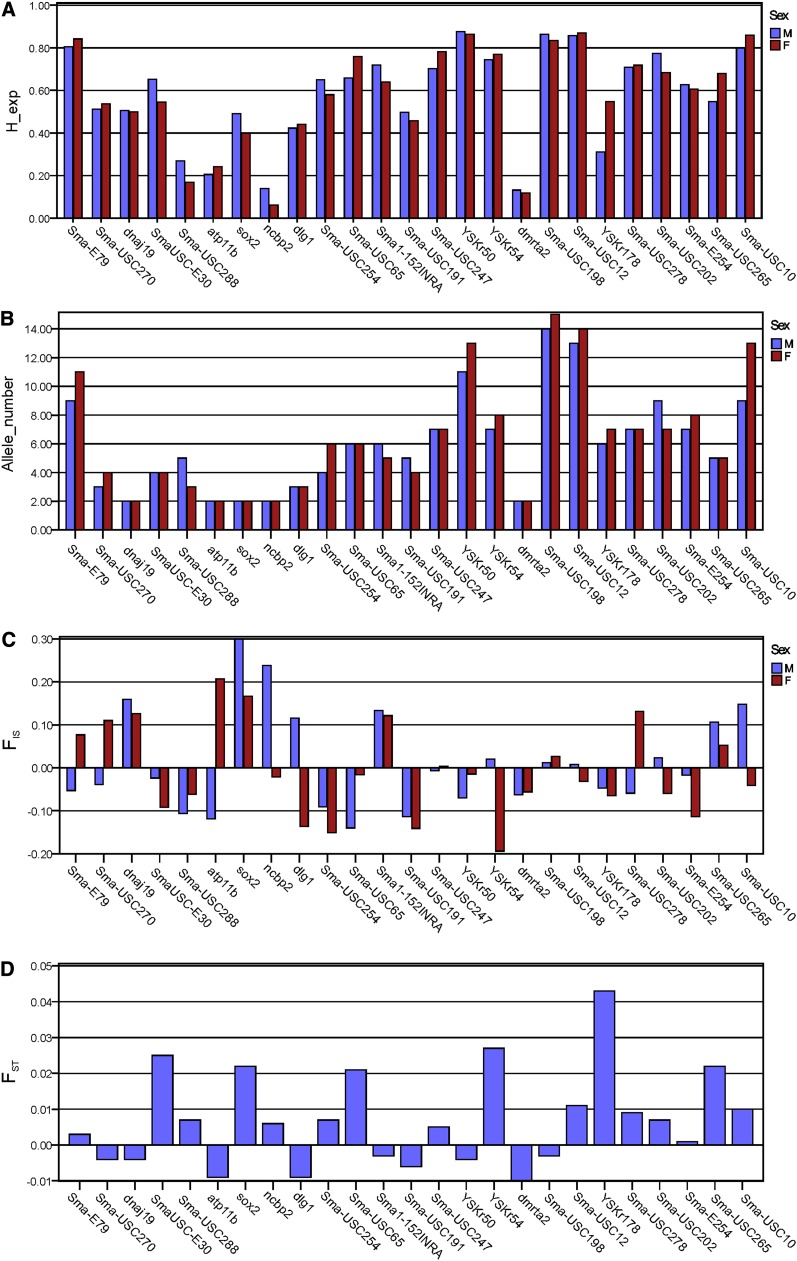
Genetic diversity along linkage group 5 in males and females of a turbot (s. Maximus) wild population. (A) expected heterozygosity (h_exp), (B) allele number (allele_number), (C) fis (within population inbreeding coefficient) and (D) fst (relative component of genetic differentiation between populations).

We evaluated genetic diversity on LG5 in a wild population of 48 males and 48 females, using 28 markers covering 71.2 cM between Sma-E79 and Sma-USC10 (Figure 1). Two markers, *fkbp2* and Sma-SNP46, were discarded because of very low diversity (H_e_ < 0.01) and one (ScmM1) because of doubtful genotyping, leaving a density of one marker per 2.8 cM in LG5, and one per 0.4 cM in the SD region between SmaUSC-E30 and SmaSNP_31 (Figure 1). Neither H_e_ nor A differed between males and females (Mann-Whitney test *P* = 0.900 and 0.803, respectively; Figure 4, A and B). The average genetic diversity, however, was significantly less in the proximal than the distal region for all the markers (H_e_: 0.473 *vs.* 0.689; A: 4.000 *vs.* 8.182 Mann-Whitney tests: *P* = 0.002 and 0, respectively) (Figure 4, A and B), or when only microsatellites were considered (proximal *vs.* distal regions: H_e_ 0.567 *vs.* 0.723, *P* = 0.004; A: 5.826 *vs.* 8.708, *P* = 0.005).

If a differentiated nonrecombinant sex-associated region exists, genetic divergence between males and females is expected, and the heterogametic sex should show greater frequencies of heterozygotes, so this is predicted for females if the turbot has a ZZ/ZW system. If, however, the W chromosome is genetically degenerated, with a high frequency of null alleles, apparently homozygous females will be found. Our analysis of F_IS_ showed only weak deviations from Hardy–Weinberg genotype proportions (*P* < 0.05) in the distal LG5 region (YSKr54 and Sma-USC198) (Figure 4C), none of them significant after Bonferroni correction. No deviations were detected within the SD region between SmaUSC-E30 and SmaSNP_31. Six significant (*P* < 0.05) F_ST_ values were detected between males and females, but none remained after Bonferroni correction, even in the main SD region, including the greatest sex-associated marker, SmaUSC-E30 (*P* = 0.127) (Figure 4D).

The analysis of LG5 genetic associations with sex in the turbot wild population rendered a picture concordant with the F_ST_ values (see Table S3), with few significant results, and none after Bonferroni correction. This finding contrasts with the significant associations within families for a large set of markers around the main SD region (*P* = 0 in family DF either at genotype or allele level, and in QF6 at the genotypic level).

### Evolution of the SD region from a closely related species

Several microsatellites from the turbot sex-associated genomic regions cross-amplified in brill (*S. rhombus*). Four such LG5 microsatellites were selected, two in the main SD region (SmaUSC-E30 and Sma-USC270) and other two in the distal one (YSKr50 and YSKr54) close to *dmrta2* and *amh*, two genes related to gonad development. Five markers, covering 23.4 cM (Sma-USC117, *sox9*, YSKr165, YSKr107, and Sma-USC231), also were selected from LG21. No LG5 markers but three LG21 microsatellites (YSKr165, *sox9*, and Sma-USC231) showed significant associations with sex in the brill population (*P* < 0.05). The greatest associations (*P* = 0.001 and 0.004 at allele and genotype level, respectively) was found at *sox9*, and differentiation between males and females was also detected (F_ST_ = 0.0561, *P* < 0.01). Also, a highly significant genotypic disequilibrium (*P* = 0) was observed between most LG21 marker pairs in males (*P* = 0: seven tests; 0.05 < *P* < 0.01: one test; *P* > 0.05: 1 test), but none in females (all tests *P* > 0.05).

## Discussion

### Genetic architecture of SD in turbot

Fish often show very small differentiated SD regions, and SD is often affected by both genetic and environmental factors (Penman and Piferrer 2008; Kikuchi and Hamaguchi 2013). In *Fugu rubripes*, for instance, only a single nucleotide in the receptor II of the anti-Mullerian hormone (*amhrII*) differentiates males and females, and in several species the differentiated SD region is less than a few kilobases (Piferrer *et al.* 2012). To date, five different master genes have been reported in fish, including rainbow trout (*sdY*; Yano *et al.* 2013), fugu (*amhrII*; Kamiya *et al.* 2012), and Patagonian pejerrey (*amhY*; Hattori *et al.* 2012), which are not close relatives but belong to different orders, and also different systems are found in closely related species like *Oryzias latipes* (*dmY*; Matsuda *et al.* 2002) and *O. luzonensis* (*gsdf1*; Myosho *et al.* 2012). On the other hand, the same SDg has been identified in two different *Oryzias* species (Matsuda *et al.* 2003), and another gene appears to be a SD gene common to most salmonids (*sdY*), suggesting SD gene jumping via mobile elements because they are located in nonhomologous genomic regions (Yano *et al.* 2013). This list may soon be enlarged, however, because a broad diversity of nonhomologous SD genomic regions has been reported within the most studied fish groups, including the *Oryzias* (medaka) genus (Tanaka *et al.* 2007), the *Gasterosteidae* (stickleback) family (Ross *et al.* 2009), the cichlid fish tilapia, in the tribe Tilapini (Cnaani *et al.* 2008), the *Salmoniformes* (salmonids) order (Phillips *et al.* 2001), and the *Poeciliidae* (guppy and platypfish) family (Tripathi *et al.* 2009).

Within flatfish, sex-associated markers have been documented in species of the family *Pleuronectidae* (*Hippoglossus hippoglossus*, Palaiokostas *et al.* 2013; *Verasper variegatus*, Ma *et al.* 2010) and *Cynoglossidae* (*Cynoglossus semilaevis*; Chen *et al.* 2014), in addition to turbot (*Scophthalmidae*). None of the other flatfish sex-associated LGs matched those in turbot using comparative mapping with model genomes as a bridge (turbot LG5 is homologous to the stickleback LGVIII, medaka LG4, fugu LG20, and Tetraodon LG1, whereas turbot LG21 is homologous to stickleback LGV and medaka LG19; Bouza *et al.* 2012) suggesting that the turbot SDg evolved independently. Remarkably, the sex-associated markers of some flatfish species from different families (*e.g.*, *C. semilaevis* and *V. variegatus*) match the same orthologous chromosomes in model teleosts syntenic to turbot autosomal LG12 (Bouza *et al.* 2012). Recently, *dmrt1* has been reported as the putative SD gene in *C. semilaevis*, which has a ZZ/ZW system and apparently a dosage compensation mechanism similar to birds (Chen *et al.* 2014). This gene at the top of the male differentiation cascade has been recurrently recruited as SD switching gene in different vertebrate groups (Mawaribuchi *et al.* 2012). Certain genes appear to be more prone to be selected when SD systems evolve, as previously suggested (Graves and Peichel 2010).

In this study, six annotated genes were consistently identified in the turbot transcriptomic database (E-value < 10^−50^) and mapped in the main SD region within a 3.0 cM-interval between the markers SmaUSC-E30 and SmaSNP_31, which defined a syntenic region to LGVIII in stickleback (Bouza *et al.* 2012). The gene order in the turbot consensus map was very similar to that in the four Acanthopterygii model fish genomes (see Table S4). Among the six candidate genes that map within the SDg region, *sox2* has been previously related to gonad differentiation (Cnaani *et al.* 2007; Mazzuchelli *et al.* 2011) and associated GO terms suggest that *dnajc19* could also be involved (GO:0000003: reproduction; GO:0022414: reproductive process; GO:0048806: genitalia development). The lack of association with sex in the natural population suggests, however, that they are not the master SDg.

Although the identification of the SDg failed in turbot, some relevant candidate genes were discarded as SDg, and the increase of marker density at the SD region provided more detailed information on the genomic region where the SDg is located and on the genetic basis of SD in turbot. New segregation data confirmed a ZZ/ZW system with a much greater number of markers than in previous reports (Martínez *et al.* 2009) and also the existence of other minor genetic or environmental factors responsible of the few discordances observed between genetic markers and phenotypic sex. Interestingly, the six discordances observed (3.9%) corresponded to males with a female genotype in both families analyzed, which could be related to incomplete dominance of a new ZZ/ZW SD system (see *Origin and evolution of SD in turbot*). The small mapping region screened regarding family sample sizes together with the lack of full congruence between phenotypes and genotypes, however, makes it difficult to precise the SDg location using our segregation approach.

Available data support that SD behaves like a complex trait in turbot, as shown in other fish. Classical quantitative genetic approaches demonstrated a polygenic SD system in European sea bass (*Dicentrarchus labrax*) and zebrafish (*Danio rerio*) (Vandeputte *et al.* 2007; Liew *et al.* 2012), and sex ratio heritability was estimated in Nile tilapia (Lozano *et al.* 2013). Also, genomic screening using genetic maps has revealed multiple genetic factors involved in SD in fish other than turbot (Bradley *et al.* 2011; Eshel *et al.* 2012; Luhmann *et al.* 2012). The quantitative nature of SD in fish could explain the turnover of SD systems, because genetic variation may be available in populations at loci involved in gonad differentiation, and this could allow turnover scenarios such as ones that have been modeled (van Doorn and Kirkpatrick 2010; Blaser *et al.* 2011).

### Origin and evolution of SD in turbot

In turbot, our marker set did not detect recombination suppression in the main SD region. Furthermore, the absence of genetic differentiation (F_ST_) between males and females or heterozygote excess in females at genes in the LG5 SD region suggests that the turbot either has no differentiated ZZ/ZW region on the chromosome pair, or that the region stopped recombining very recently. As in many other fish species (Penman and Piferrer 2008), WW individuals in turbot are viable and are used by breeders to obtain all-female populations (D. Chavarrías, personal communication). Another recurrent event during the evolution of SD chromosome pairs is the accumulation of repetitive DNA and the degeneration of the SD gene-bearing chromosome (W or Y) by deleterious alleles (Schartl 2004; Volff *et al.* 2007). Accumulation or repetitive elements has been observed in most fish species where a fine analysis of the SD region was carried out (Nanda *et al.* 1992; Peichel *et al.* 2004; Nagahama 2005), although deleterious mutation has been more rarely reported (Martínez *et al.* 2008). In the turbot LG5, we found no null alleles at microsatellite loci that might suggest degeneration of the W chromosome. However, with our much greater number of markers than in previous studies (Martínez *et al.* 2009), we found significantly reduced genetic diversity in the proximal region of this chromosome that may be related to a recent evolutionary origin. Furthermore, in previous comparative mapping (Martínez *et al.* 2009; Bouza *et al.* 2012), only a small fraction of syntenic markers to stickleback LGVIII (two of eight, 25%) and even lower to other model Acantoptherygii (12.5%, Tetraodon and Fugu) were located in the LG5 proximal region (60.7% of the whole LG5), suggesting a recent origin (or a greater evolutionary rate) for the proximal region where the SD turbot region is located.

All our data support a SD region with low genetic differentiation and of recent origin in turbot. To test this hypothesis, we cross-amplified genetic markers from the most relevant sex-associated regions of turbot in a closely related species, the brill. LG5 and LG21 were selected for this study because sex-related QTL and relevant candidate genes (LG5: *sox2*, *dnajc19*, *dmrta2*, and *amh*; LG21: *sox9* and *sox17*; Viñas *et al.* 2012; this study) were detected in these LGs, and additionally recombination was nearly suppressed in males at LG21 (Bouza *et al.* 2012). Strong associations with sex were observed with several LG21 markers, especially *sox9*, in a natural population of brill, but not for those on LG5. Also, highly significant linkage disequilibrium (*P* = 0) was detected between most LG21 markers in the brill males, but not in females, suggesting (i) lower or suppressed recombination in males across a large region that corresponds to 23.4 cM in the homologous turbot LG (58.7% LG21 length), and, furthermore (ii) an evolved XX/XY system in brill. It is interesting to note that hybrids between turbot and brill produce mostly monosex progenies whose sex depends on the sex of the turbot parent (Purdom 1976; Purdom and Thacker 1980). Although our data are preliminary, this could be related to opposite SD mechanisms and with the dominance of the recent turbot ZZ/ZW system over the older XX/XY in brill. Similar transitions either from ZZ/ZW to XX/XY or vice versa have been described in different fish groups (Cnaani *et al.* 2008; Ross *et al.* 2009; Ser *et al.* 2010).

Our segregation and association analysis has provided new information on the genetic basis and evolution of the SD system in turbot. However, this approach has shown limitations for the identification of the SDg, likely because of the small differentiated SD region and also because SD seems to behave like a complex trait in turbot. Future research should be done to refine the analysis of the SD region using the available turbot genome (Figueras *et al.* unpublished data), taking advantage of the ZZ and WW individuals obtained through hormone sex reversal parents. Additionally, comparative genomics with the new information on flatfish will provide a comprehensive vision of the evolution of SD in this relevant fish group.

## Supplementary Material

Supporting Information
