# Supplementary material for: Fine Mapping and Evolution of the Major Sex Determining Region in Turbot (Scophthalmus maximus)
Source: G3 (Bethesda). 2014 Aug 7;4(10):1871–80. doi: 10.1534/g3.114.012328 (PMC4199694; doi:10.1534/g3.114.012328)
Supplement: Supporting Information [file supp_4_10_1871__index.html]

Fine Mapping and Evolution of the Major Sex Determining Region in Turbot (Scophthalmus maximus) — Fine Mapping and Evolution of the Major Sex Determining Region in Turbot (Scophthalmus maximus) — Supporting Information 

# Fine Mapping and Evolution of the Major Sex Determining Region in Turbot (*Scophthalmus maximus*)

## Supporting Information for Taboada *et al.*, 2014

**Files in this Data Supplement:**

- Supporting Information - Figure S1 and Tables S1-S4 (PDF, 206 KB)
- Figure S1 - Crossing-over along LG5 in turbot (*S. maximus*) families. (PDF, 183 KB)
- Table S1 - Genes between the homologous turbot sequences of SmaUSC-E30 and SmaSNP\_31, at 6.06 Mb and 6.64 Mb, respectively, in the stickleback LGVIII chromosome. (PDF, 118 KB)
- Table S2 - Primer pairs and gene regions selected for developing genetic markers at putative genes in the main turbot SD region. (PDF, 150 KB)
- Table S3 - Statistical association between candidate gene-associated markers and sex at population and family level. (PDF, 149 KB)
- Table S4 - Comparative gene order of the mapped genes at the main SD region of turbot with regard to model Acanthopterygii fish genomes. (PDF, 150 KB)
