# Supplementary material for: Fine Mapping and Evolution of the Major Sex Determining Region in Turbot (Scophthalmus maximus)
Source: G3 (Bethesda). 2014 Aug 7;4(10):1871–80. doi: 10.1534/g3.114.012328 (PMC4199694; doi:10.1534/g3.114.012328)
Supplement: Supporting Information [file supp_g3.114.012328_012328SI.pdf]

G3·Genes | Genomes | Genetics

Fine mapping and evolution of the major sex determining region in turbot (*Scophthalmus maximus*)

Taboada X,[*] Hermida M,[§] Pardo BG,[§] Piferrer F,[**] Viñas A,[*] Bouza C,[§] and Martínez P[§,1]

[*]Departamento de Genética. Facultad de Biología (CIBUS). Universidad de Santiago de Compostela.. 15782 Santiago de Compostela, Spain, [§]Departamento de Genética. Facultad de Veterinaria. Universidad de Santiago de Compostela. Campus de Lugo. 27002 Lugo, Spain, and [**]Institut de Ciències del Mar, Consejo Superior de Investigaciones Científicas (CSIC), 08003 Barcelona, Spain.

[1] Corresponding autor: Departamento de Genética, Facultad de Veterinaria, USC Campus de Lugo, 27002, Lugo, Spain. E-mail: paulino.martinez@usc.es

DOI: 10.1534/g3.114.012328

Figure S1 Crossing-over along LG5 in turbot (*S. maximus*) families. Genetic markers are ordered according to the LG5 consensus genetic map (Figure 1); M: males; F: female; S: single crossover event between Z and W chromosomes; D: double crossover; N: no crossover; Lacking genotypes have been coloured with that of the adjacent markers for a better representation of crossovers.

	**DF family**		**Sma-E79**	**SmaUSC-E30**	***atp11b***	***sox2***	***fkbpg***	***dlg1***	**Sma-USC247**	**Sma1-152INRA**	**YSKr50**	**YSKr54**	***dmrta2***	**Sma-USC12**	***amh***	**Sma-E254**	**Crossover**
		M grandfather	293295	179181	AA	CT	GG	CC	256258	094096	130136	174181	542542	334340	CT	194194	
		M grandmother	293295	179179	AA	CC	GG	CA	256260	094096	128128	174181	542542	310346	CC	194194	
		F grandfather	295310	181183	AA	CT	GA	CC	254256	092102	120130	174189	542556	330334	CC	194241	
	**Offspring sex**	F grandmother	295301	179179	GA	CT	GG	AA	252258	094096	130148	181181	542542	310332	CT	194194	
		father	**295295**	**179179**	**AA**	**CC**	**GG**	**CC**	**256260**	**096096**	**128136**	**181174**	**542542**	**340346**	**CC**	**194194**	
		mother (Z)	**310**	**181**	**A**	**T**	**A**	**C**	**256**	**92**	**130**	**189**	**556**	**334**	**C**	**241**	
		mother (W)	**295**	**179**	**G**	**C**	**G**	**A**	**258**	**96**	**148**	**181**	**542**	**310**	**T**	**194**	
P3-A16	M 1	Centromere	295310	179181	AA	CT	GA	CC	258260	096096	128148	174181	542542	310346	CT	194194	S
P3-A20	M 2	Centromere	295295	179181	AA	CT	GA	CC	256260	092096	128130	174189	542556	334346	CC	194241	S
P3-A22	M 3	Centromere	295310	179181	AA	CT	GA	CC	256256	092096	130136	181189	542556	334340	CC	194241	N
P3-A26	M 4	Centromere	295295	179179	GA	CC	GG	CA	256256	092096	130136	181189	542556	334340		194241	S
P3-A27	M 5	Centromere	295310	179181	AA	CT	GA	CC	256256	092096	130136	181189	542542	310340	CT	194194	S
P3-A34	M 6	Centromere	295310	179181	AA	CT	GA	CC	256260	092096	128130	174189	542556	334346	CC	194241	N
P3-A35	M 7	Centromere	295295	179181	AA	CT	GA	CC	256260	092096	128130	174189	542556	334346	CC	194241	S
P3-A47	M 8	Centromere	295310	179181	AA	CT	GA	CC	256258	096096	136148	181181	542542	310340	CT	194194	S
P3-A51	M 9	Centromere	295310	179181	AA	CT	GA	CC	256258	096096	136148	181181	542542	310340	CT	194194	S
P3-A56	M 10	Centromere	295310	179181	AA	CT	GA	CC	256256	092096	130136	181181	542542	310340	CT	194194	S
P3-A63	M 11	Centromere	295310	179181	AA	CT	GA	CC	256256	092096	130136	181189		334340		194241	N
P3-A69	M 12	Centromere	295310	179181		CT	GA	CC	256260	092096	128130		542556	334346		194241	N

P3-A82	M 13	Centromere	295310	179181	AA	CT	GA	CC	256256	092096	130136	181189		334340		194241	N
P3-A98	M 14	Centromere	295295	179181	AA		GA			092096				334340		194194	D
P3-A100	M 15	Centromere	295310	179181	AA	CT	GA	CC	256258	096096	136148		542542	310340	CT	194194	S
P3-A110	M 16	Centromere	295310	179181	AA	CT	GA	CC	256258	096096	130136	181181	542542	310340		194241	D
P3-A115	M 17	Centromere	295310	179181					258260	096096	128148	174181	542542	310346		194194	S
P3-A116	M 18	Centromere	295295	179181	AA	CT	GA	CC	256256	092096	130136	181189	542542	310340	CT	194194	D
P3-A119	M 19	Centromere	295310	179181		CT	GA	CC	256256	092096		181189		334340		194241	N
P3-A124	M 20	Centromere	295310	179181	AA	CT	GA	CC	256260	092096	128130	174189	542556	334346	CC	194241	N
P3-B9	M 21	Centromere	295310	179181	AA	CT	GA	CC	256260	092096	128130	174189	542556	334346	CC	194241	N
P3-B13	M 22	Centromere	295310	179181	AA	CT	GA	CC	256258	096096	136148	181181	542542	310340	CT	194194	S
P3-B15	M 23	Centromere	295310	179181	AA		GA	CC	256260	092096		174189	542556	334346		194241	N
P3-B18	M 24	Centromere	295310	179181	AA	CT	GA	CC	256256	092096	130136	181189	542556	334340	CC	194241	N
P3-B19	M 25	Centromere	295310	179181	AA	CT	GA	CC	256258	096096	136148	181181	542542	310340	CT	194194	S
P3-B21	M 26	Centromere	295310	179181		CT	GA	CC	256256	092096	130136	181189	542556	334340		194241	N
P3-B28	M 27	Centromere	295295	179181	AA	CT	GA	CC	256260	092096		174189	542556	334346	CC	194241	S
P3-B30	M 28	Centromere	295295	179181	AA	CT	GA	CC	256256	092096	130136	181189	542556	334340		194241	S
P3-B38	M 29	Centromere	295310	179181	AA	CT	GA	CC	256256	092096	130136	181189	542556	334340	CC	194241	N
P3-A22	M 3	Centromere	295310	179181	AA	CT	GA	CC	256256	092096	130136	181189	542556	334340	CC	194241	N
P3-B140	M 30	Centromere	295295	179179	GA	CC	GG	CA	256256	092096	130136	181189	542556	334340	CC	194241	S
P3-B144	M 31	Centromere	295295	179181	AA	CT	GA		256256	092096	130136	181189		334340		194241	S
P3-B145	M 32	Centromere	295310	179181	AA	CT	GA	CC	256258	096096	136148	181181		310340	CT	194194	S
P3-B146	M 33	Centromere	295295	179181	AA	CT	GA	CC	256256	092096	130136	181189	542556	334340	CC	194194	D
P3-B149	M 34	Centromere	295310	179181	AA	CT	GA		256256	092096	130136	181189	542556	334340	CC	194241	N
P3-B158	M 35	Centromere	295295	179181	AA		GA	CC	256260	092096		174189		334346	CC	194241	S
P3-B162	M 36	Centromere	295310	179181	AA	CT	GA	CC	256256	092096	130136	181189	542556	334346	CC	194241	N
P3-A1	F1	Centromere	295295	179179	GA	CC	GG	CA	256258	096096	136148	181181	542542	310340		194241	S

P3-A10	F2	Centromere	295295	179179	GA	CC	GG	CA	256260	092096	128130	174189	542556	334346	CC	194241	S
P3-A11	F3	Centromere	295295	179179	GA	CC	GG	CA	256256	092096	130136	181189		334340	CC	194241	S
P3-A12	F4	Centromere	295295	179179	GA	CC	GG	CA	258260	096096	128148	174181	542542	310346	CT	194194	N
P3-A17	F5	Centromere	295295	179179	GA		GG	CA	258260	096096	128148	174181	542542	310340	CT	194194	N
P3-A24	F6	Centromere	295310	179179	GA	CC	GG	CA	258260	096096	128148	174181	542542	310346		194194	S
P3-A25	F7	Centromere	295295	179179	GA	CC	GG	CA	256256	092096	130136	181189	542556	334340	CC	194241	S
P3-A32	F8	Centromere	295295	179179	GA	CC	GG	CA	258260	096096	128148	174181	542542	310346	CT	194194	N
P3-A49	F9	Centromere	295295	179179	GA	CC	GG	CA	258260		128148	174181		310346	CT	194194	N
P3-A55	F10	Centromere	295295	179179	GA	CC	GG	CA	256258	096096	136148	181181	542542	310340	CT	194194	N
P3-A57	F11	Centromere	295295	179179	GA	CC	GG	CA	258260	096096	128148	174181	542556	334346	CC	194241	S
P3-A61	F12	Centromere	295310	179179	GA	CC	GG	CA	258260	096096	128148	174181	542542	310346	CT	194194	S
P3-A67	F13	Centromere	295295	179179	GA		GA	CC	256256	092096	130136	181189		334340		194241	S
P3-A68	F14	Centromere	295295	179179	GA	CC	GG	CA	256256	092096	130136	181189	542556	334340	CC	194241	S
P3-A73	F15	Centromere	295295	179179	GA	CC	GG	CA	256258	096096	136148	181181	542542	310340	CT	194194	N
P3-A78	F16	Centromere	295295	179179	GA			CA	258260	096096	128148	174181	542542	310346		194194	N
P3-A79	F17	Centromere	295310	179179	GA	CC	GG	CA	256258	096096	136148	181181	542542	310340		194194	S
P3-A80	F18	Centromere	295295	179179	GA	CC	GG	CA	256258	096096	136148	181181	542542	310340	CC	194241	S
P3-A97	F19	Centromere	295295	179179	GA		GG	CA	258260	096096	128148	174181		310346		194194	N
P3-A99	F20	Centromere	295295	179179	GA	CC	GG	CA	258260	096096	128148	174181	542556	334346		194241	S
P3-A109	F21	Centromere	295295	179179	GA	CC	GG	CA	256258	096096	136148	181181	542542	310340	CT	194194	N
P3-A111	F22	Centromere	295295	179179	GA			CA	256258	096096	136148	181181	542542	310340	CC	194241	S
P3-A120(1)	F23	Centromere	295310	179179	GA	CC	GG	CA	258260	096096	128148	174181	542556	334346	CC	194241	D
P3-A120(2)	F24	Centromere		179179	GA	CC	GG	CA	256258	096096	136148	181181		310340		194194	N
P3-A122	F25	Centromere	295295	179179	GA	CC	GG	CA	256258	096096	136148	181181	542542	310340	CT	194194	N
P3-A126	F26	Centromere	295295	179179	GA	CC	GG	CA	256256	092096	130136	181189	542556	334346		194241	S
P3-A129	F27	Centromere	295295	179179	GA	CC	GG	CA	256260	092096	128130	174189	542556	334346	CC	194241	S

P3-A135	F28	Centromere	295295	179179	GA	CC	GG	CA	256258	096096	136148	181181	542542	310340	CT	194194	N
P3-B8	F29	Centromere	295295	179179	GA	CC	GG	CA	256256	092096	130136	181189	542556	334340	CC	194241	S
P3-B23	F30	Centromere	295295	179179	GA	CC	GG	CA	258260	096096	128148	174181	542542	310346	CT	194194	N
P3-B33	F31	Centromere	295310	179179	GA	CC	GG	CA	258260	096096	128148	174181	542542	310346	CT	194194	S
P3-B37	F32	Centromere	295295	179179	GA	CC	GG	CA	258260	096096	128148	174181	542542	310346	CT	194194	N
P3-B64	F33	Centromere	295295	179179		CC	GG	CA	258260	096096	128148	174181	542556	334346		194241	S
P3-B81	F34	Centromere	295295	179179	GA	CC	GG	CA	258260	096096	128148	174181	542542	310346		194194	N
P3-B114	F35	Centromere	295295	179179	GA	CC	GG	CA	258260	096096	128148	174181	542542	310346	CT	194194	N
P3-B136	F36	Centromere	295295	179179	GA	CT	GG	CA	256258	096096	136148	181181	542556	334340		194241	S
P3-B137	F37	Centromere	295295	179179	GA	CC	GG	CA	256260	092096	128130	174189		334346	CT	194194	D
P3-B138	F38	Centromere		179179	GA		GG	CA	256260	092096	128130	174189	542556	334346		194194	D
P3-B139	F39	Centromere	295295	179179		CC	GG	CA	258260	096096		174181	542542	310346		194194	N
P3-B141	F40	Centromere	295295	179181	AA	CT	GA		256260	092096	128130	174189	542556	334346		194241	S
P3-B142	F41	Centromere	295295	179179	GA	CC	GG	CA	256256	092096	136148	181189	542556	334340		194241	S
P3-B143	F42	Centromere	295295	179179	GA	CC	GG	CA	256256	092096	130136	181189	542556	334340	CC	194241	S
P3-B150	F43	Centromere	295295	179179	GA	CC	GG	CA	256256	092096	130136	181189	542556	334340		194241	S
P3-B154	F44	Centromere	295295	179179	GA	CC	GG	CA	258260	096096		174181	542542	310346	CC	194241	S
P3-B155	F45	Centromere	295295	179179	GA	CC	GG	CA	256258	096096		181181		310340	CT	194194	N
P3-B159	F46	Centromere	295295	179179	GA	CC	GG	CA	256258	096096	136148	181181		334340	CC	194241	S
P3-B160	F47	Centromere	295295	179179			GG	CA	256260	092096	128130	174189		334346		194241	S
P3-B164	F48	Centromere	295295	179179		CC	GG	CA	256258	096096	136148	181181		310340		194194	N
P3-B165	F49	Centromere	295295	179179	GA	CC	GG	CA	256256	092096	130136	181189		334340	CC	194241	S
	recombinants		16	3	3	3	4	3	19	24	21	24	26	31	23	37	S=0,529
	RF		0,189	0,035	0,039	0,040	0,049	0,038	0,226	0,286	0,273	0,293	0,388	0,365	0,426	0,435	N=0,388
																	D=0,082

QF6 family		Sma-E79	*dnaj1*	SmaUSC-E30	*dlg1*	Sma-USC65	Sma-USC247	Cross over
**Offspring sex**	M grandfather		CT	183185	CA	128128	256256	
	M grandmother							
	F grandfather	297299	CT	181183	CC		256258	
	F grandmother	295301	CT	179183	CA		256260	
	father	293303	TT	183185	CC	128128	256254	
	mother (Z)	297	T	183	C	138	258	
	mother (W)	295	C	181	A	128	256	
M1	Centromere	293297	TT	183183	CA	128128	256256	S
M2	Centromere	297303	TT	183185	CC	128138	256258	N
M3	Centromere	297303	TT	183185	CC	128138	254258	N
M4	Centromere	295303	TT	183185	CC	128138	256258	S
M5	Centromere	293297		183183	CC	128138	256256	S
M6	Centromere	293297	TT	183183	CA	128128	254256	S
M7	Centromere	293297		183185	CC	128138	254258	N
M8	Centromere	293297	TT	183183	CC	128138	254258	N
M9	Centromere	293297	TT	183183	CC	128138	256258	N
M10	Centromere	297303	TT	183185	CC	128138	256256	S
M11	Centromere	297303	TT	183185	CC	128138	256258	N
M12	Centromere	297303	TT	183185	CC	128138	256256	S
M13	Centromere	293297	TT	183183	CC	128138	256258	N
M14	Centromere	295303		181185	CA	128128	254256	S
M15	Centromere	293297		183183	CC	128138	256256	S
M16	Centromere	297303	TT	183185	CC	128138	254258	N
M17	Centromere	293297	TT	183183	CC	128138	256258	N
M18	Centromere	293297		183183	CC	128138	256256	S
M19	Centromere	293297	TT	183183	CC	128138	256256	S
M20	Centromere	297303	TT	183185	CC	128138	254258	N
M21	Centromere	293297		183183	CC	128128	256256	S
M22	Centromere	297303	TT	183185	CC	128138	254258	N
M23	Centromere	297303	TT	183185	CC	128138	254258	N
M24	Centromere	293295	CT	181183	CA	128128	256256	S
M25	Centromere	295303	CT		CA	128128	256258	S
M26	Centromere	297303		183185	CC	128138	254258	N
M27	Centromere	297303	TT	183185	CC	128138	254258	N
M28	Centromere	293297	TT	183183	CC	128138	256256	S
M29	Centromere	293297	TT	183183	CC	128138	256256	N
M30	Centromere	293297	TT	183183	CC	128128	256256	S
M31	Centromere	293295	CT	181183	CA	128128	256256	S
M32	Centromere	297303	TT	183185	CC	128128	256256	S
M33	Centromere	293297	TT	183185	CC	128138	254256	S
M34	Centromere	293297	TT	183183	CC	128138		N
M35	Centromere	297303		183185	CC	128128	256256	S
M36	Centromere	297303		183185	CC	128138	254258	N
M37	Centromere	293297	TT	183183	CA	128128	256256	S
M38	Centromere	293297	TT	183183	CC	128138	254258	N
M39	Centromere	293295	TT	183183	CC	128138	256258	S
M40	Centromere	297303	TT	183185	CA	128128	254256	S
M41	Centromere	297303	TT	183183	CC	128138	256256	S
M42	Centromere	293295	CT	181183	CA	128128	254256	S
M43	Centromere	297303	TT	183185	CC	128138	254258	N
M44	Centromere	297303	CT	181185	CA	128128	256256	S
M45	Centromere	293297	TT	183185	CC	128128	254256	S
M46	Centromere	297303	TT	183185	CC	128128	256256	S
M47	Centromere	293297	TT	183183	CC	128138	256258	N
M48	Centromere	293297	TT	183183	CC	128138	256256	S
M49	Centromere	293297	TT	183183	CC	128138	256258	N

F1	Centromere	295303		181183	CA	128128	256256	N
F2	Centromere	293295	CT	181183	CA	128128	256256	N
F3	Centromere	293295		181183	CA	128128	256256	N
F4	Centromere	295303	CT	181185	CA	128128	254256	N
F5	Centromere	295303	CT	181183	CA	128128	256256	N
F6	Centromere	295303	TT	183185	CC	128138	254258	S
F7	Centromere	295303	CT	181185	CA	128128	254256	N
F8	Centromere	295303	CT	181185	CA	128128	254258	S
F9	Centromere	293295	TT	183183	CC	128138	256258	S
F10	Centromere	297303		181185	CA	128128	254256	S
F11	Centromere	297303	CT	181185	CA	128128	254256	S
F12	Centromere	295303	TT	183183	CC	128138	256258	S
F13	Centromere	293295	CT	181183	CA	128128	256256	N
F14	Centromere	293295	CT	181183	CA	128128	256256	N
F15	Centromere	293295	CT	181183	CA	128128	256256	N
F16	Centromere	293295	CT	181185	CA	128128	254258	S
F17	Centromere	297303	CT	181185	CA	128128	254256	S
F18	Centromere	293295	CT	181183	CA	128128	256256	N
F19	Centromere	295303	CT	181185	CA	128128	256256	N
F20	Centromere	293297		181183	CA	128128	256256	S
Recombina		11	8	8	12	16	29	S=0,4
RF		0,159	0,1	0,118	0,1	0,232	0,420	N=0,
								D=0,

Table S1 Genes between the homologous turbot sequences of SmaUSC-E30 and SmaSNP_31, at 6.06 Mb and 6.64 Mb, respectively, in the stickleback LGVIII chromosome.

Ensembl Gene ID	Gene Start (bp)	Gene End (bp)	Associated Gene Name
ENSGACG00000006052	6057829	6066578	*fxr1*
ENSGACG00000006076	6068380	6070562	*dnajc19*
ENSGACG00000006099	6128772	6153394	
ENSGACG00000006100	6157588	6158556	*sox2*
ENSGACG00000006104	6308502	6339588	*atp11b*
ENSGACG00000006125	6344114	6350026	*mccc1*
ENSGACG00000006138	6356136	6360741	*kng1*
ENSGACG00000006156	6364215	6377038	*PIK3CA*
ENSGACG00000006175	6384998	6388251	*ZMAT3*
ENSGACG00000006180	6394455	6397463	
ENSGACG00000006189	6448564	6475804	*LRRC7*
ENSGACG00000006210	6485834	6533369	
ENSGACG00000006216	6549844	6552127	
ENSGACG00000006222	6557305	6563824	*mfi2*
ENSGACG00000006229	6565695	6568855	*fkbp2*
ENSGACG00000006237	6566121	6567033	
ENSGACG00000006238	6569593	6572048	*ncbp2*
ENSGACG00000006258	6590103	6632830	
ENSGACG00000006285	6647309	6655988	*cp*

Table S2 Primer pairs and gene regions selected for developing genetic markers at putative genes in the main turbot SD region.

Gene	Accession number	Primers		Amplicon size	SNPs detected
dnaj19	KJ434933	F: CATGGTGGCAGTGGGACT<br>R: TGGTTCAAACCCACCTCTGT	First exon-<br>Third exon	1,133	2
		F: GCCGTGAAGCAGATGGAG<br>R: AGAAAAGTCTGGCGCTTGTG	Second exon-<br>Third exon	1,609	2
sox2	KJ434936	F: AGGAAAGTCTCCTGGAAGGAA<br>R: CAGATGAAAAGTGGGAGACG	3´UTR	662	2
atp11b	KJ434932	F: CAACATCTGCTTCACCTCGT<br>R: GGTGTCTTCTCCCATCAGGA	26th exon –<br>27th exon	454	none
		F: GTCACGCCTTCGTCTTCTTC<br>R: GTTCCAAACGTCCAGTTTCC	27th exon –<br>28th exon	1,172	none
		F: GGAAACTGGACGTTTGGAAC<br>R: CAGCTGCACGAACACAAAGT	28th exon –<br>30th exon	952	3
		F:AGACTCATTTCTGGACGTGGA<br>R: CACCACGTCGGGAAAGAG	29th exon –<br>30th exon	418	2
dlg1	KJ434935	F: CAGGAAGAGACTCTGCTCACC<br>R: GCCATCTGGAGACGCTTTAT	Third exon –<br>Sixth exon	1,871	3
		F: TGACCTCATCTCCGAGTTCC<br>R: ATGAAAACAGCGATGGGATG	Fourth exon –<br>Sixth exon	1,273	2
		F: TGACCTCATCTCCGAGTTCC<br>R: CGTGAACCGCTTGTTCATTT	Fourth exon –<br>Seventh exon	2,010	3
fkbp2	KJ434934	F: CGAGAAGAGGAAGCTCGTCA<br>R: TGCCTTGTGGTCTTCATCAG	First exon –<br>UTR3´	1,646	10
ncbp2	KJ434931	F: GCGTTGATCAGCGACTCCTA<br>R: CAGGCCGTCTTCTTGACTTT	First exon –<br>Third exon	845	2
		F: CGTGGGGAATCTCTCCTTCT<br>R: GAGTGCCATTGACGAAGC	Second exon<br>– Fourth exon	904	none
		F: ACGTGCAGACGCAGAACAC<br>R: AGTTTACCGTAGCCGCCTC	Fourth exon –<br>Fifth exon	1,145	5

Table S3 Statistical association between candidate gene-associated markers and sex at population and family level.

	Wild population		DF family		QF6 family	
	**Genotypic**	**Allelic**	**Genotypic**	**Allelic**	**Genotypic**	**Allelic**
**Sma-E79**	0.138	0.056	**0**	**0**	-	-
**Sma-USC270**	0.322	0.331	-	-	-	-
dnaj19	0.710	0.387	-	-	**0**	**0.003**
**SmaUSC-E30**	0.074	0.113	**0**	**0**	**0**	**0.001**
**Sma-USC288**	0.202	0.222	-	-	-	-
atp11b	0.322	0.623	**0**	**0**	-	-
sox2	0.292	0.097	**0**	**0**	-	-
fkbp2	-	-	**0**	**0**	-	-
ncbp2	0.433	0.182	-	-	-	-
dlg1	0.626	0.951	**0**	**0**	**0**	**0.001**
**Sma-USC254**	0.293	0.07	-	-	-	-
**Sma-USC65**	0.070	**0.038**	-	-	**0**	**0.001**
**Sma1-152INRA**	0.375	0.237	**0**	**0.002**	-	-
**Sma-USC191**	0.493	0.226	-	-	-	-
**Sma-USC247**	0.204	0.113	**0.001**	**0**	0.180	0.248
**YSKr50**	0.266	0.738	**0.002**	**0.001**	-	-
**YSKr54**	**0.011**	**0.022**	**0.002**	**0.010**	-	-
dmrta2	0.876	0.937	0.061	0.188	-	-
**Sma-USC198**	0.216	0.647	0.053	0.044	-	-
**Sma-USC12**	0.201	0.142	0.046	0.086	-	-
**YSKr178**	0.086	**0.031**	-	-	-	-
**Sma-USC278**	0.063	**0.042**	-	-	-	-
**Sma-USC88**	-	-	-	-	-	-
**Sma-USC225**	-	-	**0**	**0**	-	-
**Sma-USC202**	0.147	0.081	-	-	-	-
amh	-	-	0.394	0.771	-	-
**Sma-E254**	0.323	0.300	0.172	0.283	-	-
**Sma-USC265**	0.080	0.064	-	-	-	-
**Sma-USC10**	0.361	0.064	**0.003**	0.236	-	-

P<0.05 in bold characters. Bonferroni correction (α=0.05; k=28; P=0.0018); - non informative markers

Table S4 Comparative gene order of the mapped genes at the main SD region of turbot with regard to model Acanthopterygii fish genomes

LG5 CONSENSUS MAP TURBOT		LGVIII STICKLEBACK		LG4 MEDAKA		LG1 TETRAODON		LG20 FUGU	
dnaj19	16.3 cM	***fxr1***	6,06 Mb	*dlg1*	19,12 Mb	*dlg1*	21,329 Mb	***fxr1***	Sc. 351: 111 Kb
fxr1 **(SmaUSC-E30)**	16.5cM	*dnaj19*	6,06Mb	*fkbp2*	19,14 Mb	*fkbp2*	21,343 Mb	*dnaj19*	Sc. 351: 124 Kb
atp11b	17.6 cM	*sox2*	6,15 Mb	*ncbp2*	19,14 Mb	*ncbp2*	21,345 Mb	*sox2*	Sc. 351: 191 Kb
sox2	17.9cM	*atp11b*	6,32 Mb	***cp***	19,30 Mb	***cp***	21,403 Mb	*atp11b*	Sc. 347: 67 Kb
fkbp2	18.7 cM	*dlg1*	6,55 Mb	*atp11b*	19,59 Mb	*atp11b*	21,551 Mb	***cp***	Sc. 55: 45 Kb
ncbp2	19.0cM	*fkbp2*	6,56 Mb	*sox2*	19,85 Mb	*sox2*	21,666 Mb	*ncbp2*	Sc. 55: 121 Kb
dlg1	19.5 cM	*ncbp2*	6,57 Mb	*dnaj19*	19,99 MbSI	*dnaj19*	21,726 Mb	*fkbp2*	Sc. 55: 125 Kb
cp **(SmaSNP31)**	19.5 cM	***cp***	6,64 Mb	***fxr1***	19,99 Mb	***fxr1***	21,728 Mb	*dlg1*	Sc.55: 139 Kb

atp11b (ATPase, class VI, type 11B), *cp (*ceruloplasmin), *dlg1* (discs, large (Drosophila) homolog 1), *dnaj19* (DnaJ (Hsp40) homolog, subfamily C, member 19), *fkbp2* (FK506 binding protein 2, 13kDa), *fxr1* (fragile X mental retardation, autosomal homolog 1), *ncbp2* (nuclear cap binding protein subunit 2, 20kDa), *sox2* (SRY (sex determining region Y)-box 2).
