# Supplementary material for: Fine Mapping and Evolution of the Major Sex Determining Region in Turbot (Scophthalmus maximus)
Source: G3 (Bethesda). 2014 Aug 7;4(10):1871–80. doi: 10.1534/g3.114.012328 (PMC4199694; doi:10.1534/g3.114.012328)
Supplement: Supporting Information [file supp_g3.114.012328_FigureS1.pdf]

Figure S1 Crossing-over along LG5 in turbot (*S. maximus*) families. Genetic markers are ordered according to the LG5 consensus genetic map (Figure 1); M: males; F: female; S: single crossover event between Z and W chromosomes; D: double crossover; N: no crossover; Lacking genotypes have been coloured with that of the adjacent markers for a better representation of crossovers.

	DF family		Sma-E79	SmaUSC-E30	*atp11b*	*sox2*	*fkbpg*	*dlg1*	Sma-USC247	Sma1-152INRA	YSKr50	YSKr54	*dmrta2*	Sma-USC12	*amh*	Sma-E254	Crossover
		M grandfather	293295	179181	AA	CT	GG	CC	256258	094096	130136	174181	542542	334340	CT	194194	
		M grandmother	293295	179179	AA	CC	GG	CA	256260	094096	128128	174181	542542	310346	CC	194194	
		F grandfather	295310	181183	AA	CT	GA	CC	254256	092102	120130	174189	542556	330334	CC	194241	
	**Offspring sex**	F grandmother	295301	179179	GA	CT	GG	AA	252258	094096	130148	181181	542542	310332	CT	194194	
		father	**295295**	**179179**	**AA**	**CC**	**GG**	**CC**	**256260**	**096096**	**128136**	**181174**	**542542**	**340346**	**CC**	**194194**	
		mother (Z)	**310**	**181**	**A**	**T**	**A**	**C**	**256**	**92**	**130**	**189**	**556**	**334**	**C**	**241**	
		mother (W)	**295**	**179**	**G**	**C**	**G**	**A**	**258**	**96**	**148**	**181**	**542**	**310**	**T**	**194**	
P3-A16	M 1	Centromere	295310	179181	AA	CT	GA	CC	258260	096096	128148	174181	542542	310346	CT	194194	S
P3-A20	M 2	Centromere	295295	179181	AA	CT	GA	CC	256260	092096	128130	174189	542556	334346	CC	194241	S
P3-A22	M 3	Centromere	295310	179181	AA	CT	GA	CC	256256	092096	130136	181189	542556	334340	CC	194241	N
P3-A26	M 4	Centromere	295295	179179	GA	CC	GG	CA	256256	092096	130136	181189	542556	334340		194241	S
P3-A27	M 5	Centromere	295310	179181	AA	CT	GA	CC	256256	092096	130136	181189	542542	310340	CT	194194	S
P3-A34	M 6	Centromere	295310	179181	AA	CT	GA	CC	256260	092096	128130	174189	542556	334346	CC	194241	N
P3-A35	M 7	Centromere	295295	179181	AA	CT	GA	CC	256260	092096	128130	174189	542556	334346	CC	194241	S
P3-A47	M 8	Centromere	295310	179181	AA	CT	GA	CC	256258	096096	136148	181181	542542	310340	CT	194194	S
P3-A51	M 9	Centromere	295310	179181	AA	CT	GA	CC	256258	096096	136148	181181	542542	310340	CT	194194	S
P3-A56	M 10	Centromere	295310	179181	AA	CT	GA	CC	256256	092096	130136	181181	542542	310340	CT	194194	S
P3-A63	M 11	Centromere	295310	179181	AA	CT	GA	CC	256256	092096	130136	181189		334340		194241	N
P3-A69	M 12	Centromere	295310	179181		CT	GA	CC	256260	092096	128130		542556	334346		194241	N

P3-A82	M 13	Centromere	295310	179181	AA	CT	GA	CC	256256	092096	130136	181189		334340		194241	N
P3-A98	M 14	Centromere	295295	179181	AA		GA			092096				334340		194194	D
P3-A100	M 15	Centromere	295310	179181	AA	CT	GA	CC	256258	096096	136148		542542	310340	CT	194194	S
P3-A110	M 16	Centromere	295310	179181	AA	CT	GA	CC	256258	096096	130136	181181	542542	310340		194241	D
P3-A115	M 17	Centromere	295310	179181					258260	096096	128148	174181	542542	310346		194194	S
P3-A116	M 18	Centromere	295295	179181	AA	CT	GA	CC	256256	092096	130136	181189	542542	310340	CT	194194	D
P3-A119	M 19	Centromere	295310	179181		CT	GA	CC	256256	092096		181189		334340		194241	N
P3-A124	M 20	Centromere	295310	179181	AA	CT	GA	CC	256260	092096	128130	174189	542556	334346	CC	194241	N
P3-B9	M 21	Centromere	295310	179181	AA	CT	GA	CC	256260	092096	128130	174189	542556	334346	CC	194241	N
P3-B13	M 22	Centromere	295310	179181	AA	CT	GA	CC	256258	096096	136148	181181	542542	310340	CT	194194	S
P3-B15	M 23	Centromere	295310	179181	AA		GA	CC	256260	092096		174189	542556	334346		194241	N
P3-B18	M 24	Centromere	295310	179181	AA	CT	GA	CC	256256	092096	130136	181189	542556	334340	CC	194241	N
P3-B19	M 25	Centromere	295310	179181	AA	CT	GA	CC	256258	096096	136148	181181	542542	310340	CT	194194	S
P3-B21	M 26	Centromere	295310	179181		CT	GA	CC	256256	092096	130136	181189	542556	334340		194241	N
P3-B28	M 27	Centromere	295295	179181	AA	CT	GA	CC	256260	092096		174189	542556	334346	CC	194241	S
P3-B30	M 28	Centromere	295295	179181	AA	CT	GA	CC	256256	092096	130136	181189	542556	334340		194241	S
P3-B38	M 29	Centromere	295310	179181	AA	CT	GA	CC	256256	092096	130136	181189	542556	334340	CC	194241	N
P3-A22	M 3	Centromere	295310	179181	AA	CT	GA	CC	256256	092096	130136	181189	542556	334340	CC	194241	N
P3-B140	M 30	Centromere	295295	179179	GA	CC	GG	CA	256256	092096	130136	181189	542556	334340	CC	194241	S
P3-B144	M 31	Centromere	295295	179181	AA	CT	GA		256256	092096	130136	181189		334340		194241	S
P3-B145	M 32	Centromere	295310	179181	AA	CT	GA	CC	256258	096096	136148	181181		310340	CT	194194	S
P3-B146	M 33	Centromere	295295	179181	AA	CT	GA	CC	256256	092096	130136	181189	542556	334340	CC	194194	D
P3-B149	M 34	Centromere	295310	179181	AA	CT	GA		256256	092096	130136	181189	542556	334340	CC	194241	N
P3-B158	M 35	Centromere	295295	179181	AA		GA	CC	256260	092096		174189		334346	CC	194241	S
P3-B162	M 36	Centromere	295310	179181	AA	CT	GA	CC	256256	092096	130136	181189	542556	334346	CC	194241	N
P3-A1	F1	Centromere	295295	179179	GA	CC	GG	CA	256258	096096	136148	181181	542542	310340		194241	S

P3-A10	F2	Centromere	295295	179179	GA	CC	GG	CA	256260	092096	128130	174189	542556	334346	CC	194241	S
P3-A11	F3	Centromere	295295	179179	GA	CC	GG	CA	256256	092096	130136	181189		334340	CC	194241	S
P3-A12	F4	Centromere	295295	179179	GA	CC	GG	CA	258260	096096	128148	174181	542542	310346	CT	194194	N
P3-A17	F5	Centromere	295295	179179	GA		GG	CA	258260	096096	128148	174181	542542	310340	CT	194194	N
P3-A24	F6	Centromere	295310	179179	GA	CC	GG	CA	258260	096096	128148	174181	542542	310346		194194	S
P3-A25	F7	Centromere	295295	179179	GA	CC	GG	CA	256256	092096	130136	181189	542556	334340	CC	194241	S
P3-A32	F8	Centromere	295295	179179	GA	CC	GG	CA	258260	096096	128148	174181	542542	310346	CT	194194	N
P3-A49	F9	Centromere	295295	179179	GA	CC	GG	CA	258260		128148	174181		310346	CT	194194	N
P3-A55	F10	Centromere	295295	179179	GA	CC	GG	CA	256258	096096	136148	181181	542542	310340	CT	194194	N
P3-A57	F11	Centromere	295295	179179	GA	CC	GG	CA	258260	096096	128148	174181	542556	334346	CC	194241	S
P3-A61	F12	Centromere	295310	179179	GA	CC	GG	CA	258260	096096	128148	174181	542542	310346	CT	194194	S
P3-A67	F13	Centromere	295295	179179	GA		GA	CC	256256	092096	130136	181189		334340		194241	S
P3-A68	F14	Centromere	295295	179179	GA	CC	GG	CA	256256	092096	130136	181189	542556	334340	CC	194241	S
P3-A73	F15	Centromere	295295	179179	GA	CC	GG	CA	256258	096096	136148	181181	542542	310340	CT	194194	N
P3-A78	F16	Centromere	295295	179179	GA			CA	258260	096096	128148	174181	542542	310346		194194	N
P3-A79	F17	Centromere	295310	179179	GA	CC	GG	CA	256258	096096	136148	181181	542542	310340		194194	S
P3-A80	F18	Centromere	295295	179179	GA	CC	GG	CA	256258	096096	136148	181181	542542	310340	CC	194241	S
P3-A97	F19	Centromere	295295	179179	GA		GG	CA	258260	096096	128148	174181		310346		194194	N
P3-A99	F20	Centromere	295295	179179	GA	CC	GG	CA	258260	096096	128148	174181	542556	334346		194241	S
P3-A109	F21	Centromere	295295	179179	GA	CC	GG	CA	256258	096096	136148	181181	542542	310340	CT	194194	N
P3-A111	F22	Centromere	295295	179179	GA			CA	256258	096096	136148	181181	542542	310340	CC	194241	S
P3-A120(1)	F23	Centromere	295310	179179	GA	CC	GG	CA	258260	096096	128148	174181	542556	334346	CC	194241	D
P3-A120(2)	F24	Centromere		179179	GA	CC	GG	CA	256258	096096	136148	181181		310340		194194	N
P3-A122	F25	Centromere	295295	179179	GA	CC	GG	CA	256258	096096	136148	181181	542542	310340	CT	194194	N
P3-A126	F26	Centromere	295295	179179	GA	CC	GG	CA	256256	092096	130136	181189	542556	334346		194241	S
P3-A129	F27	Centromere	295295	179179	GA	CC	GG	CA	256260	092096	128130	174189	542556	334346	CC	194241	S

P3-A135	F28	Centromere	295295	179179	GA	CC	GG	CA	256258	096096	136148	181181	542542	310340	CT	194194	N
P3-B8	F29	Centromere	295295	179179	GA	CC	GG	CA	256256	092096	130136	181189	542556	334340	CC	194241	S
P3-B23	F30	Centromere	295295	179179	GA	CC	GG	CA	258260	096096	128148	174181	542542	310346	CT	194194	N
P3-B33	F31	Centromere	295310	179179	GA	CC	GG	CA	258260	096096	128148	174181	542542	310346	CT	194194	S
P3-B37	F32	Centromere	295295	179179	GA	CC	GG	CA	258260	096096	128148	174181	542542	310346	CT	194194	N
P3-B64	F33	Centromere	295295	179179		CC	GG	CA	258260	096096	128148	174181	542556	334346		194241	S
P3-B81	F34	Centromere	295295	179179	GA	CC	GG	CA	258260	096096	128148	174181	542542	310346		194194	N
P3-B114	F35	Centromere	295295	179179	GA	CC	GG	CA	258260	096096	128148	174181	542542	310346	CT	194194	N
P3-B136	F36	Centromere	295295	179179	GA	CT	GG	CA	256258	096096	136148	181181	542556	334340		194241	S
P3-B137	F37	Centromere	295295	179179	GA	CC	GG	CA	256260	092096	128130	174189		334346	CT	194194	D
P3-B138	F38	Centromere		179179	GA		GG	CA	256260	092096	128130	174189	542556	334346		194194	D
P3-B139	F39	Centromere	295295	179179		CC	GG	CA	258260	096096		174181	542542	310346		194194	N
P3-B141	F40	Centromere	295295	179181	AA	CT	GA		256260	092096	128130	174189	542556	334346		194241	S
P3-B142	F41	Centromere	295295	179179	GA	CC	GG	CA	256256	092096	136148	181189	542556	334340		194241	S
P3-B143	F42	Centromere	295295	179179	GA	CC	GG	CA	256256	092096	130136	181189	542556	334340	CC	194241	S
P3-B150	F43	Centromere	295295	179179	GA	CC	GG	CA	256256	092096	130136	181189	542556	334340		194241	S
P3-B154	F44	Centromere	295295	179179	GA	CC	GG	CA	258260	096096		174181	542542	310346	CC	194241	S
P3-B155	F45	Centromere	295295	179179	GA	CC	GG	CA	256258	096096		181181		310340	CT	194194	N
P3-B159	F46	Centromere	295295	179179	GA	CC	GG	CA	256258	096096	136148	181181		334340	CC	194241	S
P3-B160	F47	Centromere	295295	179179			GG	CA	256260	092096	128130	174189		334346		194241	S
P3-B164	F48	Centromere	295295	179179		CC	GG	CA	256258	096096	136148	181181		310340		194194	N
P3-B165	F49	Centromere	295295	179179	GA	CC	GG	CA	256256	092096	130136	181189		334340	CC	194241	S
	recombinants		16	3	3	3	4	3	19	24	21	24	26	31	23	37	S=0,529
	RF		0,189	0,035	0,039	0,040	0,049	0,038	0,226	0,286	0,273	0,293	0,388	0,365	0,426	0,435	N=0,388
																	D=0,082

QF6 family		Sma-E79	*dnaj1*	SmaUSC-E30	*dlg1*	Sma-USC65	Sma-USC247	Cross over
**Offspring sex**	M grandfather		CT	183185	CA	128128	256256	
	M grandmother							
	F grandfather	297299	CT	181183	CC		256258	
	F grandmother	295301	CT	179183	CA		256260	
	father	293303	TT	183185	CC	128128	256254	
	mother (Z)	297	T	183	C	138	258	
	mother (W)	295	C	181	A	128	256	
M1	Centromere	293297	TT	183183	CA	128128	256256	S
M2	Centromere	297303	TT	183185	CC	128138	256258	N
M3	Centromere	297303	TT	183185	CC	128138	254258	N
M4	Centromere	295303	TT	183185	CC	128138	256258	S
M5	Centromere	293297		183183	CC	128138	256256	S
M6	Centromere	293297	TT	183183	CA	128128	254256	S
M7	Centromere	293297		183185	CC	128138	254258	N
M8	Centromere	293297	TT	183183	CC	128138	254258	N
M9	Centromere	293297	TT	183183	CC	128138	256258	N
M10	Centromere	297303	TT	183185	CC	128138	256256	S
M11	Centromere	297303	TT	183185	CC	128138	256258	N
M12	Centromere	297303	TT	183183	CC	128138	256256	S
M13	Centromere	293297	TT	183183	CC	128138	256258	N
M14	Centromere	295303		181185	CA	128128	254256	S
M15	Centromere	293297		183183	CC	128138	256256	S
M16	Centromere	297303	TT	183185	CC	128138	254258	N
M17	Centromere	293297	TT	183183	CC	128138	256258	N
M18	Centromere	293297		183183	CC	128138	256256	S
M19	Centromere	293297	TT	183183	CC	128138	256256	S
M20	Centromere	297303	TT	183185	CC	128138	254258	N
M21	Centromere	293297		183183	CC	128128	256256	S
M22	Centromere	297303	TT	183185	CC	128138	254258	N
M23	Centromere	297303	TT	183185	CC	128138	254258	N
M24	Centromere	293295	CT	181183	CA	128128	256256	S
M25	Centromere	295303	CT		CA	128128	256258	S
M26	Centromere	297303		183185	CC	128138	254258	N
M27	Centromere	297303	TT	183185	CC	128138	254258	N
M28	Centromere	293297	TT	183183	CC	128138	256256	S
M29	Centromere	293297	TT	183183	CC	128138	256256	N
M30	Centromere	293297	TT	183183	CC	128128	256256	S
M31	Centromere	293295	CT	181183	CA	128128	256256	S
M32	Centromere	297303	TT	183185	CC	128128	256256	S
M33	Centromere	293297	TT	183185	CC	128138	254256	S
M34	Centromere	293297	TT	183183	CC	128138		N
M35	Centromere	297303		183185	CC	128128	256256	S
M36	Centromere	297303		183185	CC	128138	254258	N
M37	Centromere	293297	TT	183183	CA	128128	256256	S
M38	Centromere	293297	TT	183183	CC	128138	254258	N
M39	Centromere	293295	TT	183183	CC	128138	256258	S
M40	Centromere	297303	TT	183185	CA	128128	254256	S
M41	Centromere	297303	TT	183183	CC	128138	256256	S
M42	Centromere	293295	CT	181183	CA	128128	254256	S
M43	Centromere	297303	TT	183185	CC	128138	254258	N
M44	Centromere	297303	CT	181185	CA	128128	256256	S
M45	Centromere	293297	TT	183185	CC	128128	254256	S
M46	Centromere	297303	TT	183185	CC	128128	256256	S
M47	Centromere	293297	TT	183183	CC	128138	256258	N
M48	Centromere	293297	TT	183183	CC	128138	256256	S
M49	Centromere	293297	TT	183183	CC	128138	256258	N

F1	Centromere	295303		181183	CA	128128	256256	N
F2	Centromere	293295	CT	181183	CA	128128	256256	N
F3	Centromere	293295		181183	CA	128128	256256	N
F4	Centromere	295303	CT	181185	CA	128128	254256	N
F5	Centromere	295303	CT	181183	CA	128128	256256	N
F6	Centromere	295303	TT	183185	CC	128138	254258	S
F7	Centromere	295303	CT	181185	CA	128128	254256	N
F8	Centromere	295303	CT	181185	CA	128128	254258	S
F9	Centromere	293295	TT	183183	CC	128138	256258	S
F10	Centromere	297303		181185	CA	128128	254256	S
F11	Centromere	297303	CT	181185	CA	128128	254256	S
F12	Centromere	295303	TT	183183	CC	128138	256258	S
F13	Centromere	293295	CT	181183	CA	128128	256256	N
F14	Centromere	293295	CT	181183	CA	128128	256256	N
F15	Centromere	293295	CT	181183	CA	128128	256256	N
F16	Centromere	293295	CT	181185	CA	128128	254258	S
F17	Centromere	297303	CT	181185	CA	128128	254256	S
F18	Centromere	293295	CT	181183	CA	128128	256256	N
F19	Centromere	295303	CT	181185	CA	128128	256256	N
F20	Centromere	293297		181183	CA	128128	256256	S
Recombina		11	8	8	12	16	29	S=0,4
RF		0,159	0,1	0,118	0,1	0,232	0,420	N=0,
								D=0,
