# Supplementary material for: Fine Mapping and Evolution of the Major Sex Determining Region in Turbot (Scophthalmus maximus)
Source: G3 (Bethesda). 2014 Aug 7;4(10):1871–80. doi: 10.1534/g3.114.012328 (PMC4199694; doi:10.1534/g3.114.012328)
Supplement: Supporting Information [file supp_g3.114.012328_TableS1.pdf]

Table S1 Genes between the homologous turbot sequences of SmaUSC-E30 and SmaSNP_31, at 6.06 Mb and 6.64 Mb, respectively, in the stickleback LGVIII chromosome.

Ensembl Gene ID	Gene Start (bp)	Gene End (bp)	Associated Gene Name
ENSGACG00000006052	6057829	6066578	*fxr1*
ENSGACG00000006076	6068380	6070562	*dnajc19*
ENSGACG00000006099	6128772	6153394	
ENSGACG00000006100	6157588	6158556	*sox2*
ENSGACG00000006104	6308502	6339588	*atp11b*
ENSGACG00000006125	6344114	6350026	*mccc1*
ENSGACG00000006138	6356136	6360741	*kng1*
ENSGACG00000006156	6364215	6377038	*PIK3CA*
ENSGACG00000006175	6384998	6388251	*ZMAT3*
ENSGACG00000006180	6394455	6397463	
ENSGACG00000006189	6448564	6475804	*LRRC7*
ENSGACG00000006210	6485834	6533369	
ENSGACG00000006216	6549844	6552127	
ENSGACG00000006222	6557305	6563824	*mfi2*
ENSGACG00000006229	6565695	6568855	*fkbp2*
ENSGACG00000006237	6566121	6567033	
ENSGACG00000006238	6569593	6572048	*ncbp2*
ENSGACG00000006258	6590103	6632830	
ENSGACG00000006285	6647309	6655988	*cp*
