# Supplementary material for: Fine Mapping and Evolution of the Major Sex Determining Region in Turbot (Scophthalmus maximus)
Source: G3 (Bethesda). 2014 Aug 7;4(10):1871–80. doi: 10.1534/g3.114.012328 (PMC4199694; doi:10.1534/g3.114.012328)
Supplement: Supporting Information [file supp_g3.114.012328_TableS2.pdf]

Table S2 Primer pairs and gene regions selected for developing genetic markers at putative genes in the main turbot SD region.

Gene	Accession number	Primers		Amplicon size	SNPs detected
dnaj19	KJ434933	F: CATGGTGGCAGTGGGACT<br>R: TGGTTCAAACCCACCTCTGT	First exon-<br>Third exon	1,133	2
		F: GCCGTGAAGCAGATGGAG<br>R: AGAAAAGTCTGGCGCTTGTG	Second exon-<br>Third exon	1,609	2
sox2	KJ434936	F: AGGAAAGTCTCCTGGAAGGAA<br>R: CAGATGAAAAGTGGGAGACG	3´UTR	662	2
atp11b	KJ434932	F: CAACATCTGCTTCACCTCGT<br>R: GGTGTCTTCTCCCATCAGGA	26th exon –<br>27th exon	454	none
		F: GTCACGCCTTCGTCTTCTTC<br>R: GTTCCAAACGTCCAGTTTCC	27th exon –<br>28th exon	1,172	none
		F: GGAAACTGGACGTTTGGAAC<br>R: CAGCTGCACGAACACAAAGT	28th exon –<br>30th exon	952	3
		F:AGACTCATTTCTGGACGTGGA<br>R: CACCACGTCGGGAAAGAG	29th exon –<br>30th exon	418	2
dlg1	KJ434935	F: CAGGAAGAGACTCTGCTCACC<br>R: GCCATCTGGAGACGCTTTAT	Third exon –<br>Sixth exon	1,871	3
		F: TGACCTCATCTCCGAGTTCC<br>R: ATGAAAACAGCGATGGGATG	Fourth exon –<br>Sixth exon	1,273	2
		F: TGACCTCATCTCCGAGTTCC<br>R: CGTGAACCGCTTGTTCATTT	Fourth exon –<br>Seventh exon	2,010	3
fkbp2	KJ434934	F: CGAGAAGAGGAAGCTCGTCA<br>R: TGCCTTGTGGTCTTCATCAG	First exon –<br>UTR3´	1,646	10
ncbp2	KJ434931	F: GCGTTGATCAGCGACTCCTA<br>R: CAGGCCGTCTTCTTGACTTT	First exon –<br>Third exon	845	2
		F: CGTGGGGAATCTCTCCTTCT<br>R: GAGTGCCATTGACGAAGC	Second exon<br>– Fourth exon	904	none
		F: ACGTGCAGACGCAGAACAC<br>R: AGTTTACCGTAGCCGCCTC	Fourth exon –<br>Fifth exon	1,145	5
