# Supplementary material for: Fine Mapping and Evolution of the Major Sex Determining Region in Turbot (Scophthalmus maximus)
Source: G3 (Bethesda). 2014 Aug 7;4(10):1871–80. doi: 10.1534/g3.114.012328 (PMC4199694; doi:10.1534/g3.114.012328)
Supplement: Supporting Information [file supp_g3.114.012328_TableS3.pdf]

Table S3 Statistical association between candidate gene-associated markers and sex at population and family level.

	Wild population		DF family		QF6 family	
	**Genotypic**	**Allelic**	**Genotypic**	**Allelic**	**Genotypic**	**Allelic**
**Sma-E79**	0.138	0.056	**0**	**0**	-	-
**Sma-USC270**	0.322	0.331	-	-	-	-
dnaj19	0.710	0.387	-	-	**0**	**0.003**
**SmaUSC-E30**	0.074	0.113	**0**	**0**	**0**	**0.001**
**Sma-USC288**	0.202	0.222	-	-	-	-
atp11b	0.322	0.623	**0**	**0**	-	-
sox2	0.292	0.097	**0**	**0**	-	-
fkbp2	-	-	**0**	**0**	-	-
ncbp2	0.433	0.182	-	-	-	-
dlg1	0.626	0.951	**0**	**0**	**0**	**0.001**
**Sma-USC254**	0.293	0.07	-	-	-	-
**Sma-USC65**	0.070	**0.038**	-	-	**0**	**0.001**
**Sma1-152INRA**	0.375	0.237	**0**	**0.002**	-	-
**Sma-USC191**	0.493	0.226	-	-	-	-
**Sma-USC247**	0.204	0.113	**0.001**	**0**	0.180	0.248
**YSKr50**	0.266	0.738	**0.002**	**0.001**	-	-
**YSKr54**	**0.011**	**0.022**	**0.002**	**0.010**	-	-
dmrta2	0.876	0.937	0.061	0.188	-	-
**Sma-USC198**	0.216	0.647	0.053	0.044	-	-
**Sma-USC12**	0.201	0.142	0.046	0.086	-	-
**YSKr178**	0.086	**0.031**	-	-	-	-
**Sma-USC278**	0.063	**0.042**	-	-	-	-
**Sma-USC88**	-	-	-	-	-	-
**Sma-USC225**	-	-	**0**	**0**	-	-
**Sma-USC202**	0.147	0.081	-	-	-	-
amh	-	-	0.394	0.771	-	-
**Sma-E254**	0.323	0.300	0.172	0.283	-	-
**Sma-USC265**	0.080	0.064	-	-	-	-
**Sma-USC10**	0.361	0.064	**0.003**	0.236	-	-

P<0.05 in bold characters. Bonferroni correction (α=0.05; k=28; P=0.0018); - non informative markers
