# Supplementary material for: Fine Mapping and Evolution of the Major Sex Determining Region in Turbot (Scophthalmus maximus)
Source: G3 (Bethesda). 2014 Aug 7;4(10):1871–80. doi: 10.1534/g3.114.012328 (PMC4199694; doi:10.1534/g3.114.012328)
Supplement: Supporting Information [file supp_g3.114.012328_TableS4.pdf]

Table S4 Comparative gene order of the mapped genes at the main SD region of turbot with regard to model Acanthopterygii fish genomes

LG5 CONSENSUS MAP TURBOT		LGVIII STICKLEBACK		LG4 MEDAKA		LG1 TETRAODON		LG20 FUGU	
dnaj19	16.3 cM	***fxr1***	6,06 Mb	*dlg1*	19,12 Mb	*dlg1*	21,329 Mb	***fxr1***	Sc. 351: 111 Kb
fxr1 **(SmaUSC-E30)**	16.5cM	*dnaj19*	6,06Mb	*fkbp2*	19,14 Mb	*fkbp2*	21,343 Mb	*dnaj19*	Sc. 351: 124 Kb
atp11b	17.6 cM	*sox2*	6,15 Mb	*ncbp2*	19,14 Mb	*ncbp2*	21,345 Mb	*sox2*	Sc. 351: 191 Kb
sox2	17.9cM	*atp11b*	6,32 Mb	***cp***	19,30 Mb	***cp***	21,403 Mb	*atp11b*	Sc. 347: 67 Kb
fkbp2	18.7 cM	*dlg1*	6,55 Mb	*atp11b*	19,59 Mb	*atp11b*	21,551 Mb	***cp***	Sc. 55: 45 Kb
ncbp2	19.0cM	*fkbp2*	6,56 Mb	*sox2*	19,85 Mb	*sox2*	21,666 Mb	*ncbp2*	Sc. 55: 121 Kb
dlg1	19.5 cM	*ncbp2*	6,57 Mb	*dnaj19*	19,99 MbSI	*dnaj19*	21,726 Mb	*fkbp2*	Sc. 55: 125 Kb
cp **(SmaSNP31)**	19.5 cM	***cp***	6,64 Mb	***fxr1***	19,99 Mb	***fxr1***	21,728 Mb	*dlg1*	Sc.55: 139 Kb

atp11b (ATPase, class VI, type 11B), *cp* (ceruloplasmin), *dlg1* (discs, large (Drosophila) homolog 1), *dnaj19* (DnaJ (Hsp40) homolog, subfamily C, member 19), *fkbp2* (FK506 binding protein 2, 13kDa), *fxr1* (fragile X mental retardation, autosomal homolog 1), *ncbp2* (nuclear cap binding protein subunit 2, 20kDa), *sox2* (SRY (sex determining region Y)-box 2).
